# Oxytocin and the Role of Fluid Restriction in MDMA-Induced Hyponatremia: A Secondary Analysis of 4 Randomized Clinical Trials

**DOI:** 10.1001/jamanetworkopen.2024.45278

**Published:** 2024-11-15

**Authors:** Cihan Atila, Isabelle Straumann, Patrick Vizeli, Julia Beck, Sophie Monnerat, Friederike Holze, Matthias E. Liechti, Mirjam Christ-Crain

**Affiliations:** 1 Department of Endocrinology, Diabetology and Metabolism, University Hospital Basel, Basel, Switzerland; 2 Department of Clinical Research, University of Basel, University Hospital Basel, Basel, Switzerland; 3 Division of Clinical Pharmacology and Toxicology, University Hospital Basel, Basel, Switzerland

## Abstract

**Importance:**

3,4-Methylenedioxymethamphetamine (MDMA, or ecstasy) is a recreational drug being investigated for the treatment of posttraumatic stress disorder. Acute hyponatremia is a potentially serious complication after even a single dose of MDMA. The assumed etiology has been a vasopressin release inducing the syndrome of inappropriate antidiuresis combined with increased thirst, causing polydipsia and water intoxication.

**Objective:**

To investigate the incidence and severity of hyponatremia after a single dose of MDMA, underlying neuroendocrine mechanisms of action, and the potential effect of fluid restriction on lowering the incidence of hyponatremia.

**Design, Setting, and Participants:**

This ad hoc secondary analysis pooled data from 4 placebo-controlled crossover randomized clinical trials conducted at the University Hospital Basel, Basel, Switzerland. The 96 participants received experimental doses of MDMA between March 1, 2017, and August 31, 2022.

**Intervention:**

A single oral 100- or 125-mg dose of MDMA. Fluid intake was not restricted in 81 participants; it was restricted in 15.

**Main Outcomes and Measures:**

Plasma oxytocin, copeptin (marker of vasopressin), and sodium levels were measured repeatedly within 360 minutes after MDMA intake. The association of plasma oxytocin or copeptin levels with plasma sodium level at 180 minutes (peak concentration of MDMA) was determined.

**Results:**

Among the 96 participants, the mean (SD) age was 29 (7) years, and 62 (65%) were men. A total of 39 participants (41%) received a 100-mg dose of MDMA, and 57 (59%) received a 125-mg dose. At baseline, the mean (SD) plasma sodium level was 140 (3) mEq/L and decreased in response to MDMA by 3 (3) mEq/L. Hyponatremia occurred in 30 participants (31%) with a mean (SD) sodium level of 133 (2) mEq/L. In 15 participants with restricted fluid intake, no hyponatremia occurred, while in the 81 participants with unrestricted fluid intake, hyponatremia occurred in 30 (37%) (*P* = .002) with a difference in plasma sodium of 4 (95% CI, 2-5) mEq/L (*P* < .001) between both groups, suggesting that fluid restriction may mitigate the risk of hyponatremia. At baseline, the mean (SD) plasma oxytocin level was 87 (45) pg/mL and increased in response to MDMA by 388 (297) pg/mL (ie, a mean [SD] 433% [431%] increase at 180 minutes), while the mean (SD) copeptin level was 4.9 (3.8) pmol/L and slightly decreased, by 0.8 (3.0) pmol/L. Change in plasma sodium level from baseline to 180 minutes demonstrated a negative correlation with the changes in oxytocin (*R* = −0.4; *P* < .001) and MDMA (*R* = −0.4; *P* < .001) levels while showing no correlation with the change in copeptin level.

**Conclusions and Relevance:**

In this secondary analysis of 4 randomized clinical trials, a high incidence of acute hyponatremia was observed in response to MDMA, which may be mitigated by fluid restriction. Hyponatremia was associated with acute oxytocin but not copeptin release. This challenges the current hypothesis of direct vasopressin release and rather indicates that oxytocin mimics the effect of vasopressin in the kidneys due to structural homology.

## Key Points

**Question:**

What are the incidence, severity, and underlying mechanisms of 3,4-methylenedioxymethamphetamine (MDMA)–induced hyponatremia, and can fluid restriction mitigate its occurrence?

**Findings:**

This secondary analysis of 4 randomized clinical trials including 96 participants found a 31% incidence of acute hyponatremia following administration of a single dose of MDMA, which may be effectively mitigated by fluid restriction. Hyponatremia was associated with increased oxytocin rather than copeptin release, suggesting that oxytocin mimics vasopressin’s kidney effects due to structural similarities.

**Meaning:**

These findings offer new insights into the neuroendocrine basis of MDMA-induced hyponatremia and contribute to the understanding of safety protocols for MDMA use.

## Teaser

This secondary analysis of 4 randomized clinical trials investigates the association of MDMA with activation of the oxytocin and vasopressin systems, the incidence and severity of MDMA-induced hyponatremia, and the role of fluid restriction in lowering the risk of hyponatremia.

## Introduction

3,4-Methylenedioxymethamphetamine (MDMA, also known as “ecstasy”) is a recreational drug that is also being investigated for the treatment of posttraumatic stress disorder.^1^ MDMA has been associated with multiple toxic effects, including acute hyponatremia, a potentially serious complication arising from ingesting even a single dose, and possibly consequential seizures, coma, and death due to cerebral edema.^2^ Hyponatremia results from a relative excess of total body water in relation to exchangeable sodium ions and develops when ingested water cannot be completely excreted by the kidneys.^3^

The underlying mechanisms through which MDMA leads to hyponatremia remain hypothetical. According to the existing literature, the suspected etiology is an increased vasopressin release from the posterior pituitary gland, inducing the syndrome of inappropriate antidiuresis (SIAD) combined with increased thirst, leading to polydipsia and water intoxication.^2^ More precisely, it has been suggested that MDMA’s structural resemblance to serotonin (5-HT) increases the concentration of hypothalamic serotonin and dopamine responsible for direct vasopressin release.^4^ Vasopressin leads to free water reabsorption and retention via vasopressin 2 receptors (V2R) in the kidneys, thus exacerbating the dilution of plasma sodium. In addition, research has highlighted excessive water intake due to hyperpyresia, dry mouth, and the drug’s stimulant effects in settings of prolonged physical activity, such as dancing, and crowded, warm environments such as clubs or festivals, contributing to the dilution of plasma sodium levels. In SIAD or polydipsia, fluid restriction is the first-line therapy in treating and preventing hyponatremia.^5^ To our knowledge, there are no experimental data on whether fluid restriction can prevent or lower the risk of MDMA-induced hyponatremia. Moreover, data on the incidence or severity of hyponatremia and the underlying neuroendocrine mechanisms are confined to case series or uncontrolled observational and retrospective settings.

Therefore, this secondary analysis of 4 randomized clinical trials investigated the association of MDMA with activation of oxytocin and vasopressin systems, the incidence and severity of MDMA-induced hyponatremia, and the possible role of fluid restriction in lowering the risk of hyponatremia. We primarily hypothesized a high incidence of hyponatremia in response to MDMA and that the decrease in sodium level might be more pronounced in participants without restricted fluid intake.

## Methods

### Study Design

This ad hoc pooled analysis of 4 randomized, placebo-controlled, double-blind, crossover clinical trials included 96 healthy participants. The trials were conducted between March 1, 2017, and August 31, 2022, at the University Hospital Basel, Basel, Switzerland. All trials focused on the psychoactive effects of MDMA and have previously been published (eFigure 4 in Supplement 1).^6,7,8,9^ The first study, by Holze et al,^6^ included 28 healthy participants who took part in sessions with MDMA (125 mg), lysergic acid diethylamide (LSD), amphetamine, and placebo. The second study, by Vizeli et al,^7^ included 29 healthy participants who received MDMA (125 mg) and placebo. The third study, by Atila et al,^8^ included 15 healthy participants who received MDMA (100 mg) and placebo. The fourth study, by Straumann et al,^9^ included 24 healthy participants who received MDMA (100 mg), LSD, the combination of MDMA and LSD, and a placebo. For all studies, the MDMA-only sessions were used for this analysis. The 4 studies included a total of 96 MDMA administrations. The studies conformed to the Declaration of Helsinki^10^ and were approved by the Ethics Committee Northwest Switzerland. The use of MDMA was authorized by the Swiss Federal Office for Public Health, Bern, Switzerland. All participants provided written informed consent before participating in the study and were paid for participation. The trials followed the Consolidated Standards of Reporting Trials (CONSORT) reporting guidelines. The trial protocols are all found in Supplement 2.

### Participants

All participants were screened for somatic and psychological comorbidities and only included if no somatic or psychological illnesses were present. Exclusion criteria are reported elsewhere in detail.^6,7,8,9^ The main exclusion criteria were a history of psychiatric disorders or psychotic disorders in first-degree relatives (assessed by the Semi-Structured Clinical Interview for *Diagnostic and Statistical Manual of Mental Disorders*, 5th edition, Axis I disorders); physical illness; the use of medication that may interfere with the study medication (eg, any psychiatric medication); tobacco smoking (>10 cigarettes/d); lifetime prevalence of illicit substance use more than 5, 10, or 20 times (except for tetrahydrocannabinol [THC]), depending on the study; illicit drug use within the last 2 months; and illicit drug use during the study, determined by results of urine tests conducted before the test sessions.

### Study Procedure

The 4 studies included a screening visit, 2 to 4 test sessions in a randomized order, and an end-of-study visit. The washout period between the main visits lasted at least 10 days. The test sessions were conducted in a calm hospital room. Only 1 research participant and 1 investigator were present during each test session. The test sessions began at 8:00 am. A urine sample was taken to verify abstinence from drugs of abuse (opiates, cocaine, amphetamines, methamphetamines, and THC), and a urine pregnancy test was performed on participants of childbearing potential. An intravenous catheter was placed in an antecubital vein for blood sampling and kept open with minimal rate of 0.9% saline. The participants underwent baseline measurements, including vital signs and blood sampling. MDMA was administered at 9:00 am. Each experimental session lasted 7 to 13 hours, depending on the study, and participants were under continuous medical supervision until any subjective effects had completely subsided. Standardized meals (breakfast and lunch) were served, and participants were allowed to drink fluids not containing alcohol or xanthine. Participants were lying in a hospital bed most of the time and were not physically active (no dancing, etc) besides walking to the restroom.

In the first, second, and fourth studies, fluid intake was not limited. Patients were allowed fluids ad libitum, and participants received an undefined amount of intravenous 0.9% saline. In the third study, additional venous blood gas analyses were performed to measure sodium levels at the given time points, and participants received a maximum of 250 mL of intravenous 0.9% saline within 7 hours. If a decrease in the sodium level of more than 1 mEq/L was observed, restriction of fluid intake was recommended (to convert mEq/L to mmol/L, multiply by 1.0).

### Study Drugs

Oral MDMA was prepared as opaque gelatine capsules containing 25 mg of pharmaceutically pure MDMA hydrochloride (ReseaChem GmbH) with mannitol filler and administered as a single dose of 100 mg (4 capsules of 25 mg each) or 125 mg (5 capsules of 25 mg each) orally. All products were prepared and quality controlled according to Good Manufacturing Practice guidelines (Dr Hysek AG).^11^ Based on a prior pharmacokinetic study,^12^ peak effects of MDMA were expected after 2.5 hours and expected psychoactive effects of 6 hours.

### Blood Samples

Samples were collected to determine osmolality and plasma levels of oxytocin, sodium, urea, uric acid, and potassium levels at 0, 90, 120, 150, 180, 300, and 360 minutes and for plasma copeptin levels at 0, 90, 120, and 180 minutes. For all 4 studies, the given laboratory values were available at baseline and 180 minutes (ie, expected peak concentration of MDMA) and for 90-, 120-, 150-, 300-, and 360-minute differences between the studies. Samples were taken as aliquots (EDTA plasma, lithium-heparin, and serum), immediately centrifuged at 4 °C at 3000 rpm for 10 minutes, then stored at −80 °C. Osmolality and plasma levels of sodium, urea, uric acid, and potassium were measured in 1 batch. Copeptin levels were measured using a commercial automated immunofluorescence assay (B.R.A.H.M.S Copeptin-proAVP KRYPTOR; Thermo Scientific). Extracted EDTA plasma oxytocin was created using a 96-well plate with each well containing 30 mg of sorbent (Oasis PRiME HLB; Waters Corporation). For oxytocin determination, the oxytocin enzyme-linked immunosorbent assay kit (ENZO Life Sciences) (sensitivity, 15 pg/mL; range, 15.6-1000.0 pg/mL) was used. The intra-assay coefficient of variation for oxytocin measurements is 1.59%, and the interassay coefficient of variation is 4.97%. The antiserum displays cross-reactivity with mesotocin of 7%, arginine vasotocin of 7.5%, and less than 0.02% for other related molecules. MDMA levels were determined in plasma using high-performance liquid chromatography–tandem mass spectrometry. The lower limit of quantification of MDMA was 0.5 ng/mL. A validated bioanalytical method was used for the analysis.^13^ Pharmacokinetic parameters were estimated using noncompartmental methods in Phoenix WinNonlin, version 8.3 (Certara).

### Objectives

There were 2 main objectives. First, we computed the incidence and severity of hyponatremia within 360 minutes after a single oral dose of MDMA. Second, we investigated the association of fluid restriction on plasma sodium levels and the association of MDMA on the vasopressinergic (by measuring plasma copeptin levels) and oxytocinergic (by measuring plasma oxytocin levels) systems.

### Statistical Analysis

Demographic information was described as mean (SD) or absolute (relative) frequency. All plasma laboratory values after MDMA intake were described by mean (SD) for baseline and 180 minutes, and the time course was visualized using the mean (SD). First, for the incidence and severity of hyponatremia, the lowest plasma sodium level from MDMA intake to the end of the session (360 minutes) was assessed for each participant, and plasma sodium levels of less than 135 mEq/L were defined as hyponatremia and described by absolute (relative) frequency. Second, Pearson correlation between changes (from baseline to 180 minutes) in plasma sodium level with (1) plasma oxytocin levels, (2) plasma MDMA levels, and (3) plasma copeptin levels was assessed. In case of significant correlation, the effect of plasma oxytocin or copeptin on plasma sodium level at 180 minutes (peak concentration of MDMA) was analyzed using a linear regression model. The models included plasma sodium levels at 180 minutes as a dependent continuous variable, plasma oxytocin or copeptin levels at 180 minutes as independent variables, and further adjustments for baseline sodium level, sex, body mass index (BMI; calculated as the weight in kilograms divided by the height in meters squared), and MDMA dose as covariates. Third, the association of unrestricted fluid intake vs restricted fluid intake with plasma sodium level was analyzed using a linear regression model, including plasma sodium levels at 180 minutes as a dependent continuous variable, group (participants with unrestricted vs restricted fluid intake) as independent variable, and further adjustments for baseline sodium level, sex, BMI, and MDMA dose as covariates. We also conducted a Fisher exact test to compare the proportions of hyponatremia between the groups with and without fluid restriction. No data imputation was foreseen for missing data, and only the full statistical analysis set was used. All analyses were performed in R, version 4.2.3 (R Program for Statistical Computing). Two-sided *P* < .05 indicated statistical significance.

## Results

### Baseline Characteristics

In total, 96 healthy adult participants were included in this analysis. The mean (SD) age was 29 (7) years; 34 participants (35%) were women and 62 (65%) were men. A total of 39 participants (41%) received an MDMA dose of 100 mg and 57 (59%) received an MDMA dose of 125 mg. In the group with no fluid intake restriction (n = 81), the mean (SD) age was 28 (5) years, 26 participants (32%) were women, and 55 (68%) were men. In the group with restricted fluid intake (n = 15), the mean (SD) age was 36 (11) years, 8 of 15 participants (53%) were women, and 7 (47%) were men. Baseline characteristics are summarized in Table 1.

**Table 1.** Baseline Characteristics of Study Participants

Characteristic	Pooled data (n = 96)	Fluid intake restriction	
		None (n = 81)	Present (n = 15)
Sex, No. (%)			
Female	34 (35)	26 (32)	8 (53)
Male	62 (65)	55 (68)	7 (47)
Age, mean (SD), y	29 (7)	28 (5)	36 (11)
Weight, mean (SD), kg	71 (10)	72 (10)	70 (10)
Height, mean (SD), cm	175 (7)	176 (8)	173 (10)
BMI, mean (SD)	23.1 (2.3)	23.1 (2.4)	23.2 (2.1)
MDMA dose, No. (%)			
100 mg	39 (41)	24 (30)	15 (100)
125 mg	57 (59)	57 (70)	0

Abbreviations: BMI, body mass index (calculated as the weight in kilograms divided by the height in meters squared); MDMA, 3,4-methylenedioxymethamphetamine.

### Plasma Sodium Level Change in Response to MDMA

After MDMA intake, mean plasma MDMA levels peaked at 180 minutes with 226 (50) ng/mL. The mean maximum plasma level during the experimental session was 245 (54) ng/mL. No differences in plasma MDMA levels were observed between participants without and with restricted fluid intake at 180 minutes, with mean levels of 233 (59) ng/mL and 189 (42) ng/mL, respectively.

**Figure 1.**
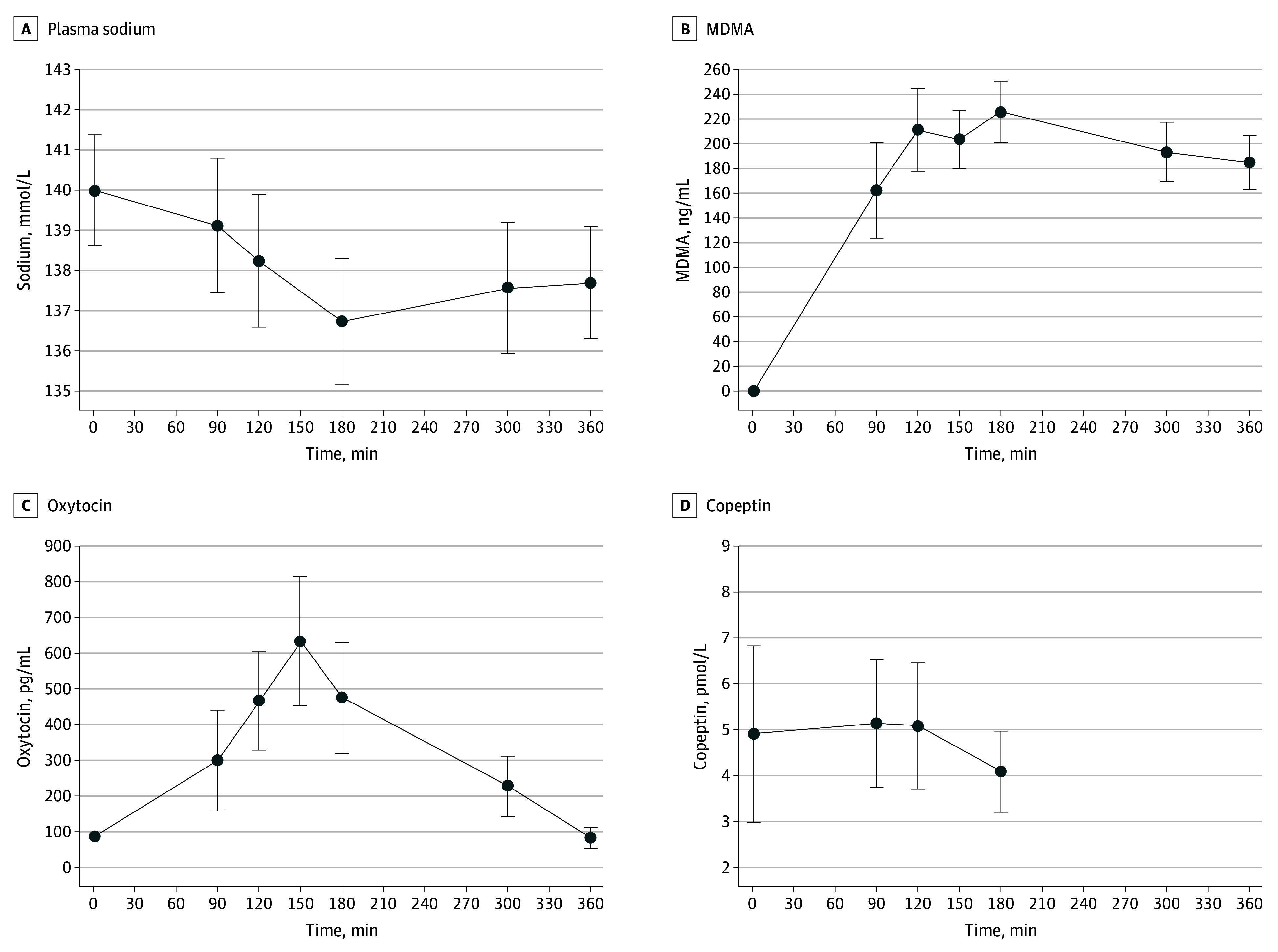
Changes in Laboratory Values Over 360 Minutes in Response to 3,4-Methylenedioxymethamphetamine (MDMA) Intake Data are expressed as mean (SD) in 96 participants.

Changes in plasma sodium levels from baseline (ie, directly before study drug intake) and the time to peak MDMA level are demonstrated in Table 2, with sex differences in the eTable in Supplement 1. The time course of changes in plasma sodium levels after MDMA intake is shown in Figure 1.

**Table 2.** Laboratory Results at Baseline and 180 Minutes After MDMA Intake in Study Participants^a^

Plasma level	Pooled data (N = 96)	Fluid intake restriction	
		None (n = 81)	Present (n = 15)
Sodium, mEq/L			
Baseline	140 (3)	140 (3)	141 (1)
180 min	137 (3)	136 (3)	140 (2)
Change at 180 min	−3 (3)	−4 (3)	−1 (2)
Maximum change	−4 (2)	−5 (2)	−2 (2)
MDMA, ng/mL			
Baseline	0 (0)	0 (0)	0 (0)
180 min	226 (50)	233 (49)	189 (42)
Change at 180 min	226 (50)	233 (49)	189 (42)
Maximum change	245 (54)	251 (53)	214 (47)
Oxytocin, pg/mL			
Baseline	87 (45)	88 (46)	79 (35)
180 min	474 (309)	452 (294)	598 (367)
Change at 180 min	387 (297)	364 (277)	519 (368)
Maximum change	501 (314)	481 (308)	609 (336)
Copeptin, pmol/L			
Baseline	4.9 (3.8)	5.0 (3.9)	4.3 (3.5)
180 min	4.1 (1.8)	4.2 (1.8)	3.4 (1.4)
Change at 180 min	−0.8 (3.0)	−0.8 (3.1)	−0.9 (3.0)
Maximum change	0.4 (3.5)	0.5 (3.6)	−0.3 (2.8)
Osmolality, mOsm/L			
Baseline	289 (7)	289 (7)	291 (5)
180 min	284 (7)	283 (7)	290 (6)
Urea, mg/dL			
Baseline	12.6 (4.2)	12.9 (4.5)	11.5 (3.4)
180 min	10.9 (3.4)	10.9 (3.4)	10.9 (3.1)
Uric acid, mg/dL			
Baseline	4756.3 (1243.7)	4806.7 (1260.5)	4470.6 (1159.7)
180 min	4403.4 (1058.8)	4437.0 (1025.2)	4252.1 (1327.7)
Glucose, mg/dL			
Baseline	97.3 (16.2)	95.5 (16.2)	108.1 (9.0)
180 min	100.9 (14.4)	100.9 (14.4)	108.1 (14.4)
Potassium, mEq/L			
Baseline	4.0 (0.5)	4.0 (0.6)	4.0 (0.3)
180 min	4.0 (0.5)	4.0 (0.5)	4.1 (0.5)

Abbreviation: MDMA, 3,4-methylenedioxymethamphetamine.

SI conversion factors: To convert glucose to mmol/L, multiply by 0.0555; osmality to mmol/kg, multiply by 1.0; potassium to mmol/L, multiply by 1.0; sodium to mmol/L, multiply by 1.0; urea to mmol/L, multiply by 0.357; uric acid to mmol/L, multiply by 0.0595.

^a^ Values are expressed as mean (SD).

At baseline, the mean (SD) plasma sodium level was 140 (3) mEq/L and decreased in response to MDMA by 3 (3) mEq/L. Hyponatremia occurred in 30 of 96 participants (31%) after intake of MDMA. Among participants with hyponatremia, the mean sodium level was 133 (2) mEq/L, and no profound hyponatremia was observed (eFigure 2 in Supplement 1). The decrease in plasma sodium level at the peak concentration of MDMA (ie, 180 minutes) was associated with a higher dose of MDMA (125 vs 100 mg: −1 [95% CI, −3 to 1] mEq/L; *P* = .045 [N = 96]) and lower BMI (per 1 unit: −0.2 [95% CI, −1 to 0] mEq/L; *P* = .04 [N = 96]), but no sex differences (women vs men: 0 [95% CI, −1 to 0] mEq/L; *P* = .87 [N = 96]) were observed (eFigure 3 in Supplement 1).

In participants without fluid intake restriction, the mean (SD) baseline plasma sodium level was 140 (3) mEq/L and decreased in response to MDMA by 4 (3) mEq/L. In contrast, in participants with fluid restriction, the mean (SD) baseline plasma sodium level was 141 (1) mEq/L and decreased slightly in response to MDMA by 1 (2) mEq/L. Participants who did not restrict fluid intake had a significantly higher incidence of hyponatremia (30 of 81 [37%]) compared with those with restricted fluid intake (0 of 15) (*P* = .002). In the subgroup receiving an MDMA dose of 100 mg (n = 39), participants who did not restrict fluid intake had a significantly higher incidence of hyponatremia (9 of 24 [38%]) compared with those with restricted fluid intake (0 of 15) (*P* = .007). At the peak concentration of MDMA (ie, 180 minutes), fluid restriction was associated with higher plasma sodium levels (difference: 4 [95% CI, 2-5] mEq/L; *P* < .001 [N = 96]) in the univariate analysis. This association remained with further adjustment in the multivariable analysis (difference: 3 [95% CI, 1-5] mEq/L; *P* = .003 [N = 96]).

### Plasma Oxytocin and Copeptin Levels in Response to MDMA and Association With Plasma Sodium

**Figure 2.**
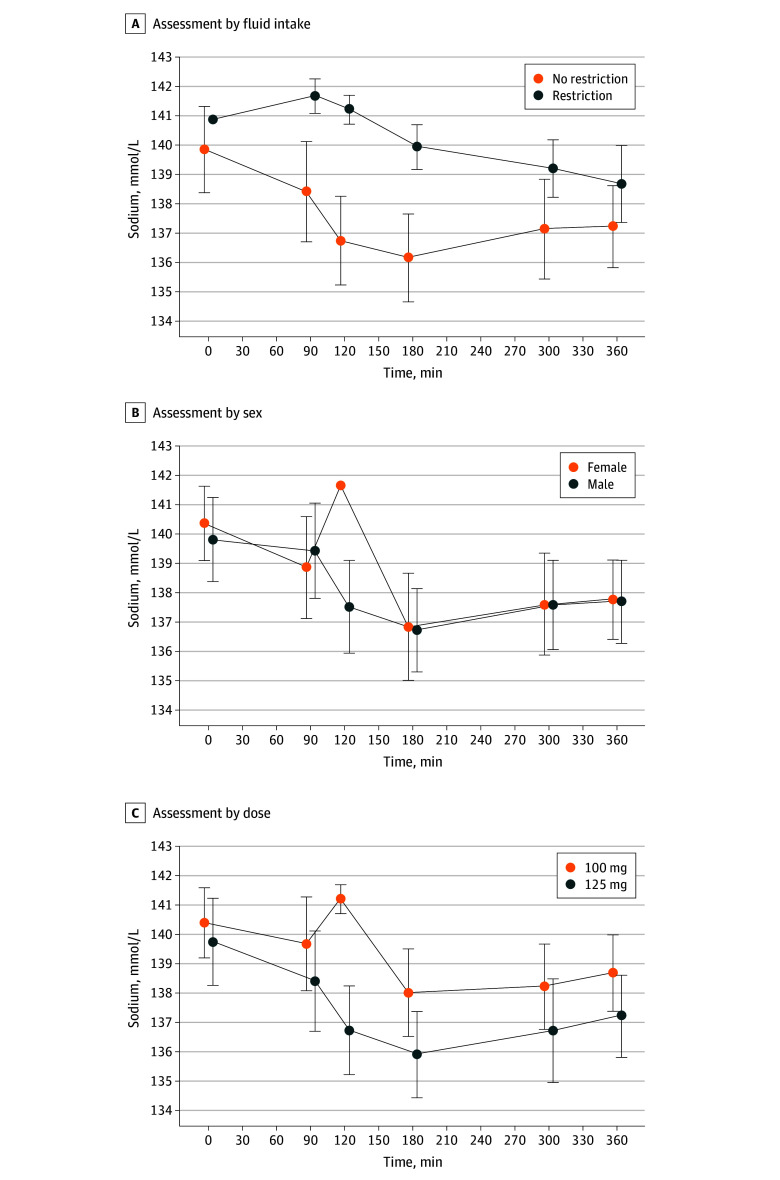
Plasma Sodium Levels in Subgroups in Response to 3,4-Methylenedioxymethamphetamine (MDMA) Intake Data are expressed as mean (SD) in 96 participants.

Changes in plasma oxytocin and copeptin levels from baseline and the time to peak level are demonstrated in Table 2, with sex differences in the eTable in Supplement 1. The time course of change in plasma oxytocin and copeptin levels after MDMA intake is shown in Figure 2.

**Figure 3.**
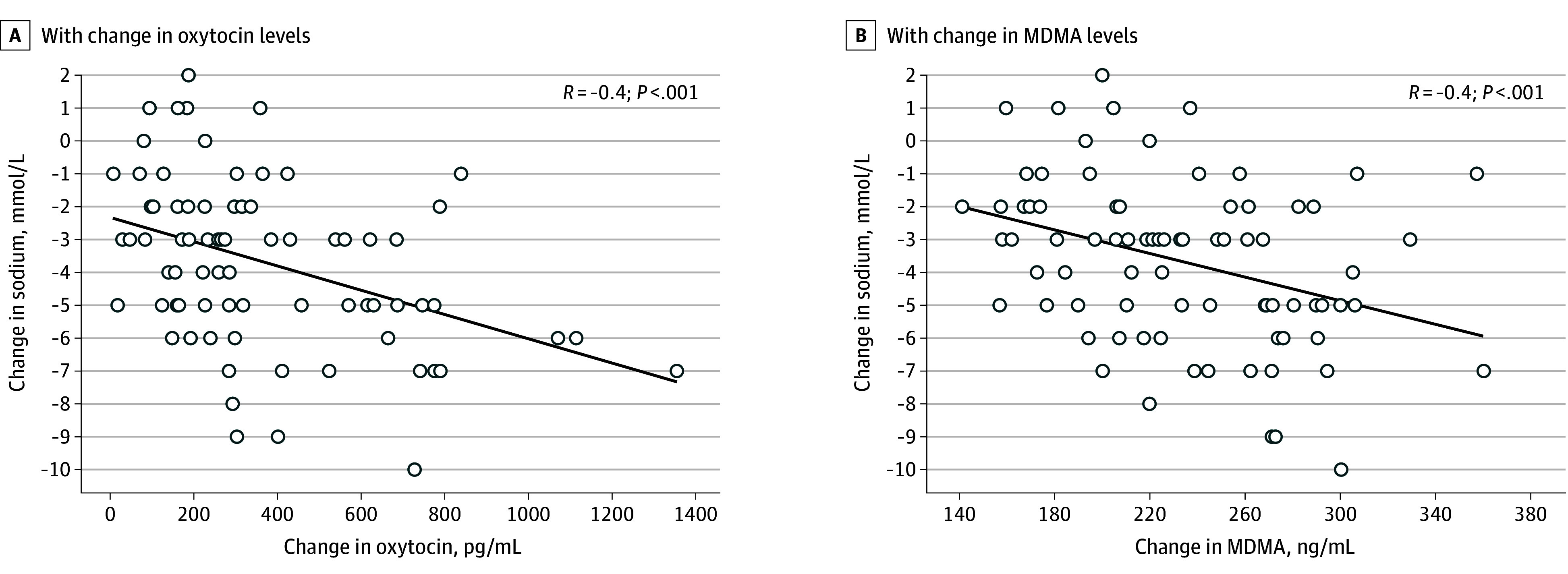
Correlation in the Change in Plasma Sodium With the Change in Plasma Oxytocin and 3,4-Methylenedioxymethamphetamine (MDMA) Levels Data are expressed as a scatterplot in 81 participants who did not have restricted fluid intake. Each dot represents 1 observation per participant assessing the change from baseline to 180 minutes with a correlation line and is described with a Pearson correlation coefficient.

At baseline, the mean (SD) plasma oxytocin level was 87 (45) pg/mL and increased in response to MDMA by 388 (297) pg/mL to 474 (309) pg/mL at 180 minutes, resulting in a mean (SD) increase of 433% (431%) (Figure 1). At baseline, the mean plasma copeptin level was 4.9 (3.8) pmol/L and slightly decreased in response to MDMA by 0.8 (3.0) pmol/L to 4.1 (1.8) pmol/L at 180 minutes. In participants without restricted fluid intake, there was a negative correlation between the change in sodium level from baseline to 180 minutes and the change in plasma oxytocin levels (*R* = −0.4; *P* < .001) and change in plasma MDMA levels (*R* = −0.4; *P* < .001) while showing no correlation with the change in copeptin levels (*R* = 0.1; *P* = .53) (Figure 3).

At 180 minutes, in participants without restricted fluid intake, a decrease in plasma sodium level was associated with increasing plasma oxytocin levels (100-pg/mL increase in oxytocin: −0.3 [95% CI, −0.5 to −0.1] mEq/L; *P* = .003 [N = 96]) in the univariable analysis. There was no association with changes in plasma copeptin level (1-pmol/L increase in copeptin level: −0.1 [95% CI, −0.2 to 0.1] mEq/L; *P* = .59 [N = 96]). With further adjustment in the multivariable analysis, the association of plasma oxytocin with sodium level persisted (−0.2 [95% CI, −0.5 to 0] mEq/L; *P* = .03 [N = 96]).

### Plasma Osmolality and Urea, Uric Acid, Glucose, and Potassium Levels in Response to MDMA

Changes from baseline and the time to peak level are demonstrated in Table 2, and sex differences are shown in eTable in Supplement 1. The time course of plasma osmolality, and changes in levels of urea, uric acid, and potassium after MDMA intake are shown in eFigure 1 in Supplement 1.

While a decreases in plasma osmolality and urea and uric acid levels were observed, no association of MDMA was shown with plasma glucose and potassium levels. In participants without restricted fluid intake, the decreases in plasma osmolality and urea and uric acid levels were slightly stronger than in those with restricted fluid intake.

## Discussion

This pooled secondary analysis of 4 randomized clinical trials of controlled MDMA administrations has 3 main findings. First, we report a high incidence of acute hyponatremia in response to MDMA. Second, our data suggest that fluid restriction may effectively prevent hyponatremia. Third, hyponatremia was associated with acute strong oxytocin but no copeptin increase, challenging the current hypothesis of vasopressin release and rather indicating that the increase in oxytocin level mimics the effect of vasopressin in the kidneys due to close structural similarities.

Acute MDMA-induced hyponatremia was first described by Maxwell et al^14^ in 1993, and since then, several cases or case series have been published.^15,16,17^ In these reports, hyponatremia presented as a life-threatening complication arising from the recreational use of MDMA with a plasma sodium level at admission ranging from 101 to 130 mEq/L.^2^ However, recording the exact incidence and severity of MDMA-induced hyponatremia is challenging. Herein, we provide controlled data from an experimental study cohort, demonstrating that despite the administered doses of MDMA being within a safe range and in a controlled setting, we observed a high incidence of 37% of hyponatremia in participants who did not restrict their fluid intake. The mean decrease in plasma sodium level was modest, with 4 (3) mEq/L, similar to other smaller MDMA studies.^12,18,19,20^ We suspect the true incidence in a club setting to be higher and the decrease more pronounced, since users might consume higher or often unknown doses of MDMA in an environment favoring the intake of large hydration volumes and in the possible presence of physical activity (dancing). A retrospective analysis of the California Poison Control System reported a hyponatremia (<130 mEq/L) prevalence of 38.8% and a 4-fold odds for hyponatremia and related coma in women.^21^ This same study reported 4 deaths due to cerebral edema in 73 patients with hyponatremia (3 women and 1 man). In contrast to these data, we observed no sex difference for the incidence of hyponatremia but an association with lower BMI and, in agreement with this, higher plasma MDMA concentration in women compared with men. Nevertheless, it is important to note that premenopausal women with hyponatremia have an increased risk for hyponatremic encephalopathy, which is thought to be due to estrogen-dependent cerebral adaptation to hypo-osmolality.^13,22^ Additional sex differences in MDMA pharmacokinetics or higher plasma concentrations of MDMA in women compared with men might partially be attributed to this observation.^23^ In support of this, a Dutch prospective observational study^24^ reported a hyponatremia prevalence of 14.3% in 63 MDMA users at a rave party, with a much higher prevalence in women (26.7%) compared with men (3.0%) despite taking the same number of pills, probably due to lower baseline sodium level, body weight, and BMI and proportionally higher body fat and lower body water percentage in women.

The suspected main underlying etiology of hyponatremia is an increased vasopressin release inducing SIAD. However, most evidence is based on reports from clubbing settings, and only a few smaller controlled studies are available, with inconsistent results regarding vasopressin release.^12,18,19,25^ While some, including the previous study by Simmler et al,^18^ have demonstrated increased vasopressin levels with MDMA intake—although mostly marginally increased—others could not confirm this.^12,18,19,20,26,27^ Surprisingly, in one study,^28^ plasma MDMA and vasopressin levels even demonstrated a negative correlation, which is the opposite of what would be predicted for vasopressin-induced SIAD. Contrary to these conflicting observations on vasopressin release, animal and human data consistently show its strong effect on oxytocin.^4,29,30,31^ This is also supported by our results, demonstrating a 5-fold increase in oxytocin level with MDMA administration. Although the exact mechanisms of oxytocin release have not been fully elucidated, MDMA has been shown to activate oxytocinergic neurons with a high density of 5-HT_1A_ receptors. In line with this, the administration of 5-HT_1A_ agonists increases oxytocin levels in rats without affecting vasopressin levels.^29,32^

Importantly, our data revealed relevant correlations between increasing oxytocin levels and decreasing plasma sodium levels, but there was no correlation of MDMA with copeptin level. While oxytocin is primarily recognized for its crucial role in childbirth and lactation, limited data indicates additional involvement in regulating water balance. Available evidence is primarily based on case reports in the context of labor, where the administration of high oxytocin doses is associated with a higher risk of hyponatremia.^33,34,35^ In experimental settings, oxytocin administration increases urinary aquaporin-2 levels, suggesting an antidiuretic role via V2R-mediated tubular aquaporin-2 insertion.^36,37^ This functional interaction with binding affinity and crosstalk at V2R likely arises from structural similarities between oxytocin and vasopressin, as both only differ by 2 amino acids.^38,39,40^ Oxytocin’s affinity for V2R in the kidneys can be observed, especially at supraphysiological levels, as induced by MDMA. This is therefore most likely explaining the antidiuretic effect in our cohort.^41^ Thus, our data suggest an oxytocin-mediated hyponatremia following MDMA intake rather than a vasopressin effect, challenging the current hypothesis of direct vasopressin release as the primary cause of MDMA-induced hyponatremia. Vasopressin secretion reported in some cases might be influenced by stress, physical activity, and nicotine use—all of which are common exposures in clubbing environments—and may contribute as a confounding factor rather than the main effect.^42,43^

In SIAD, fluid restriction is the first-line therapy in treating and preventing hyponatremia.^5^ We herein demonstrate that fluid restriction was associated with higher plasma sodium levels, leading to no case of hyponatremia. Therefore, these findings suggest that fluid intake should be restricted when MDMA is used in therapeutic settings, such as in MDMA-assisted therapy for posttraumatic stress disorder. An additional factor exacerbating the decrease in plasma sodium level with MDMA administration involves dry mouth and increased thirst perception, leading to polydipsia supported by a study by Simmler et al^18^ demonstrating a significant increase in oral fluid intake following MDMA intake, doubling within 6 hours compared with placebo. In support of this, a small study of 12 participants showed that oral water loading of 20 mL/kg within 30 minutes in addition to MDMA resulted in a greater decrease of plasma sodium level than in addition to placebo.^20^ Polydipsia observed upon MDMA intake may be attributed to an increase in body temperature, changes in the central drive to drink, and also the misconception emphasizing the need to drink to avoid dehydration.^44^ Acute adverse effects like dry mouth, physical exertion in club settings with elevated ambient temperatures, and subsequent hyperhydration with electrolyte-free water may further contribute. Overall, contrary to previous recommendations for “providing plenty of water at parties,” caution against excessive intake of hypotonic fluids is advised in recreational settings.

### Strengths and Limitations

Our study has strengths. Overall, our findings provide novel insights into the neuroendocrine mechanisms underlying MDMA-induced hyponatremia. This analysis used studies conducted in a rigorously standardized and controlled setting, mitigating the influence of confounding factors. Moreover, our analysis provides valuable safety data concerning MDMA use.

Our study also has some limitations. The study sample size in the fluid restriction group was small and sex imbalance between the groups should be noted. Only single doses of MDMA were used, and no placebo control was assessed. Furthermore, the total amount of fluid intake and intravenous saline application was only recorded in patients performing fluid restriction. Most participants without restriction of fluid intake had a slightly higher dose of MDMA; however, we adjusted our results on the MDMA dose, mitigating this limitation. Finally, no urinalysis was performed in our studies to recognize the SIAD pattern.

## Conclusions

In this secondary analysis of 4 randomized clinical trials, a high incidence of acute hyponatremia was observed in response to MDMA, which may be mitigated by fluid restriction. These findings suggest that the high incidence of acute hyponatremia in response to a single dose of MDMA may be mediated by oxytocin rather than vasopressin and may be mitigated by fluid restriction.

## Notes

Supplement 1. **eFigure 1.** Laboratory Measures in Response to MDMA Intake **eFigure 2.** Individual Plasma Sodium Levels at Baseline and the Minimum Plasma Level Throughout the Experimental Session After MDMA Intake for Each Participant **eFigure 3.** Plasma Measurements in Women and Men **eTable.** Laboratory Results **eFigure 4.** Study Flow Diagram

Supplement 2.
Trial Protocols

Supplement 3.
Data Sharing Statement
