# Supplementary material for: Oxytocin and the Role of Fluid Restriction in MDMA-Induced Hyponatremia: A Secondary Analysis of 4 Randomized Clinical Trials
Source: JAMA Netw Open. 2024 Nov 15;7(11):e2445278. doi: 10.1001/jamanetworkopen.2024.45278 (PMC11568463; doi:10.1001/jamanetworkopen.2024.45278)
Supplement: Supplement 1. — eFigure 1. Laboratory Measures in Response to MDMA Intake eFigure 2. Individual Plasma Sodium Levels at Baseline and the Minimum Plasma Level Throughout the Experimental Session After MDMA Intake for Each Participant eFigure 3. Plasma Measurements in Women and Men eTable. Laboratory Results eFigure 4. Study Flow Diagram [file jamanetwopen-e2445278-s001.pdf]

Supplementary Online Content

Atila C, Straumann I, Vizeli P, et al. Oxytocin and the role of fluid restriction in MDMA-induced hyponatremia: a secondary analysis of 4 randomized clinical trials. *JAMA Netw Open*. 2024;7(11):e2445278. doi:10.1001/jamanetworkopen.2024.45278

eFigure 1. Laboratory Measures in Response to MDMA Intake

eFigure 2. Individual Plasma Sodium Levels at Baseline and the Minimum Plasma Level Throughout the Experimental Session After MDMA Intake for Each Participant

eFigure 3. Plasma Measurements in Women and Men

eTable. Laboratory Results

eFigure 4. Study Flow Diagram

This supplementary material has been provided by the authors to give readers additional information about their work.

© 2024 Atila C et al. *JAMA Network Open*.

eFigure 1. Laboratory Measures in Response to MDMA Intake

(A) Plasma osmolality decreased in response to MDMA intake. (B) Plasma glucose levels showed no major change in response to MDMA intake. (C) Plasma urea decreased in response to MDMA intake. (D) Plasma uric acid decreased in response to MDMA intake. (E) Plasma potassium showed no major change in response to MDMA intake. Data are expressed as boxplots, N=96.

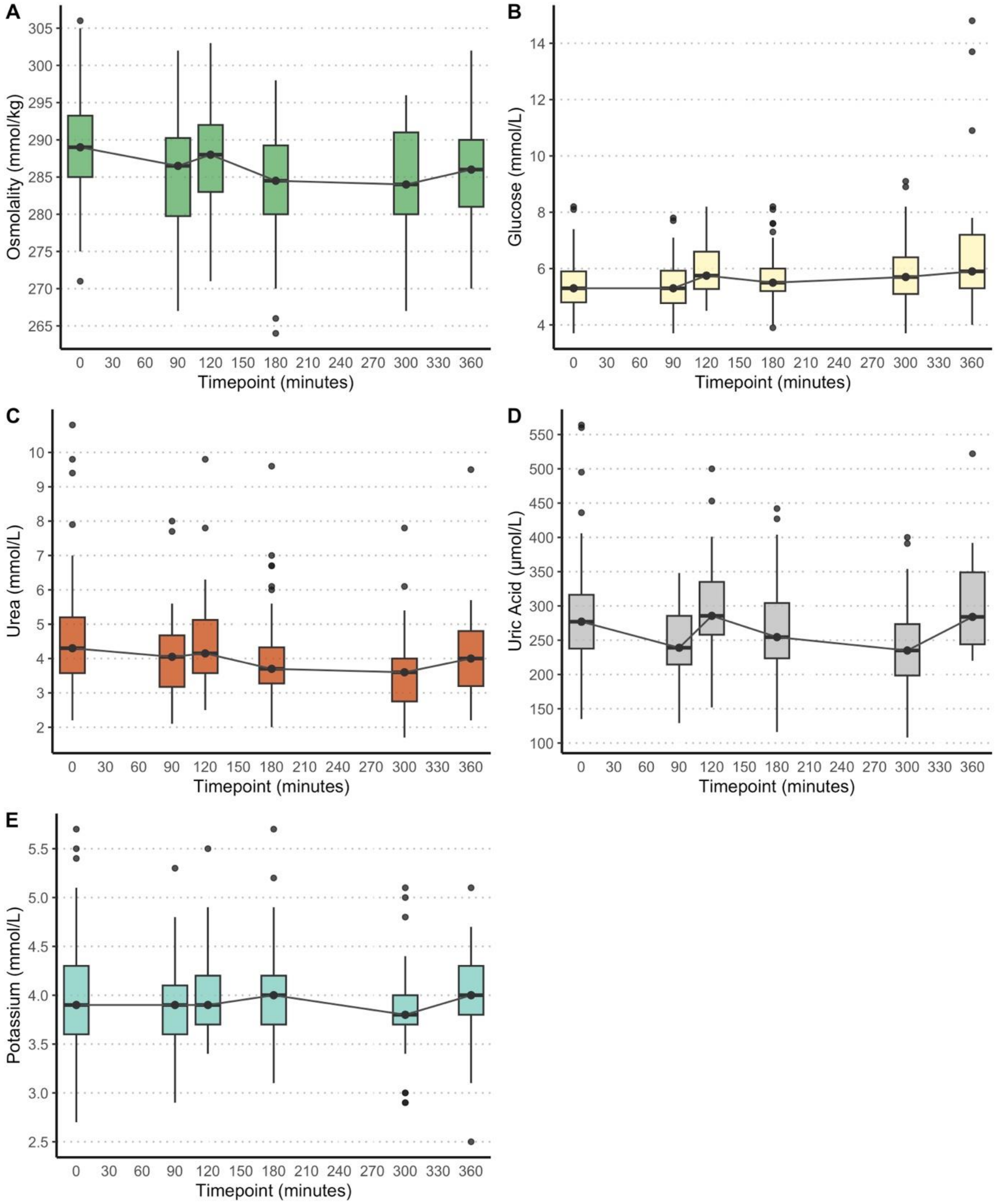

© 2024 Atila C et al. *JAMA Network Open*.

eFigure 2. Individual Plasma Sodium Levels at Baseline and the Minimum Plasma Level Throughout the Experimental Session After MDMA Intake for Each Participant

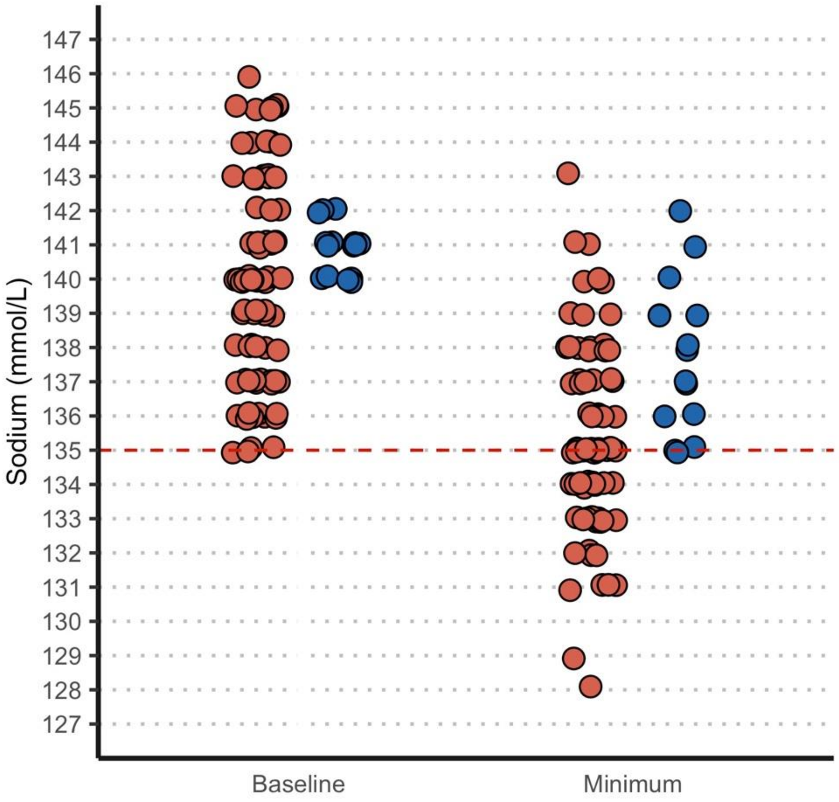

Data are expressed as individual dots in participants not restricted to fluid intake (orange) and participants restricted to fluid intake (blue), N=96.

© 2024 Atila C et al. *JAMA Network Open*.

eFigure 3. Plasma Measurements in Women and Men

(A) Plasma sodium, (C) plasma MDMA, (E) plasma oxytocin, and (G) plasma copeptin in females in response to MDMA intake. (B) Plasma sodium, (D) plasma MDMA, (F) plasma oxytocin, and (H) plasma copeptin in males in response to MDMA intake. Data are expressed as mean (standard deviation), N=34 females, N=62 males.

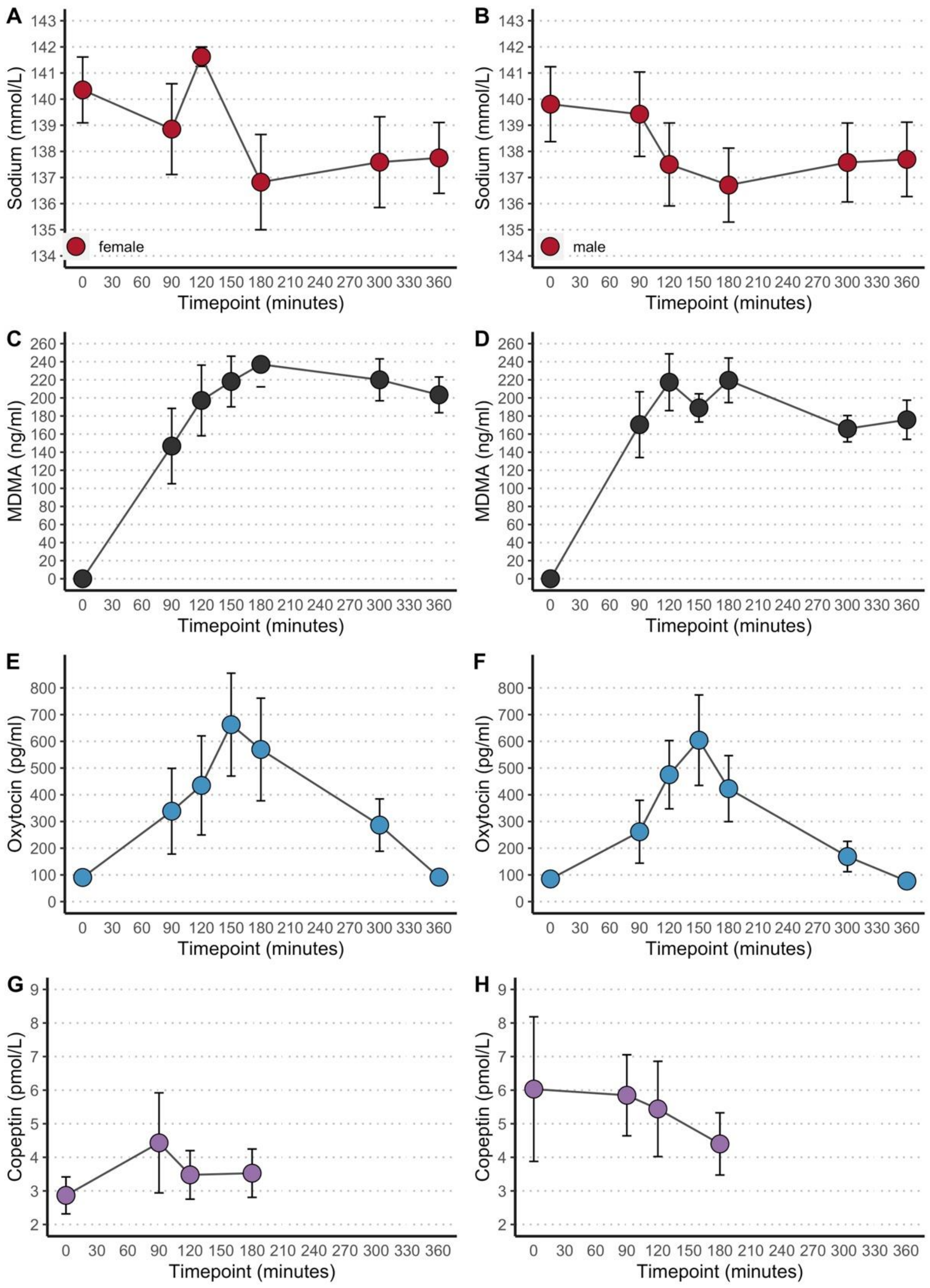

© 2024 Atila C et al. *JAMA Network Open*.

eTable. Laboratory Results

	Pooled Data Female		Pooled Data Male		No Fluid Restriction Female		No Fluid Restriction Male		Fluid restriction Female		Fluid restriction Male	
	(n=34)		(n=62)		(n=26)		(n=55)		(n=8)		(n=7)	
	**Baseline**	**180 minutes**	**Baseline**	**180 minutes**	**Baseline**	**180 minutes**	**Baseline**	**180 minutes**	**Baseline**	**180 minutes**	**Baseline**	**180 minutes**
**Plasma sodium,** mmol/l	140 (3)	137 (4)	140 (3)	137 (3)	140 (3)	136 (3)	140 (3)	136 (3)	141 (1)	141 (1)	141 (1)	139 (2)
**Change^{180min}**	-4 (3)		-3 (2)		-5 (3)		-3 (2)		0 (1)		-2 (2)	
**Change^{Max}**	-5 (3)		-4 (2)		-6 (2)		-4 (2)		-3 (2)		-3 (2)	
**Plasma MDMA,** ng/ml	0 (0)	237 (50)	0 (0)	220 (49)	0 (0)	250 (41)	0 (0)	224 (50)	0 (0)	196 (55)	0 (0)	182 (22)
**Change^{180min}**	237 (50)		220 (49)		250 (41)		224 (50)		196 (55)		182 (22)	
**Change^{Max}**	258 (52)		238 (53)		269 (46)		242 (54)		226 (60)		201 (25)	
**Plasma oxytocin,** pg/ml	90 (48)	570 (384)	85 (43)	423 (247)	94 (49)	537 (374)	86 (45)	412 (241)	80 (47)	675 (425)	78 (17)	511 (295)
**Change^{180min}**	479 (369)		338 (237)		444 (350)		326 (230)		594 (430)		433 (290)	
**Change^{Max}**	599 (355)		447 (277)		578 (351)		435 (276)		666 (385)		544 (285)	
**Plasma copeptin,** pmol/l	2.9 (1.1)	3.5 (1.4)	6.0 (4.3)	4.4 (1.9)	2.8 (0.9)	3.5 (1.4)	6.1 (4.3)	4.5 (1.9)	3.1 (1.6)	3.6 (1.7)	5.6 (4.6)	3.3 (1.2)
**Change^{180min}**	0.7 (1.1)		-1.6 (3.5)		0.7 (1.1)		-1.5 (3.4)		0.4 (1.0)		-2.4 (3.8)	
**Change^{Max}**	1.6 (2.6)		-0.3 (3.8)		1.9 (2.8)		-0.2 (3.8)		0.8 (1.4)		-1.5 (3.6)	
**Plasma osmolality,** mOsm/l	289 (6)	283 (8)	290 (7)	284 (7)	288 (6)	281 (8)	290 (7)	283 (7)	291 (5)	288 (5)	291 (6)	292 (6)
**Plasma urea,** mmol/l	4.1 (1.4)	3.5 (1.0)	4.8 (1.6)	4.2 (1.2)	4.3 (1.5)	3.5 (1.1)	4.8 (1.6)	4.1 (1.2)	3.5 (1.0)	3.3 (0.8)	4.7 (1.1)	4.5 (1.1)
**Plasma uric acid,** mmol/l	229 (46)	210 (46)	313 (69)	291 (52)	231 (48)	213 (48)	312 (71)	288 (50)	221 (39)	199 (41)	318 (58)	314 (66)
**Plasma glucose,** mmol/l	5.3 (0.8)	5.6 (0.7)	5.5 (0.9)	5.7 (0.9)	5.0 (0.7)	5.6 (0.7)	5.4 (0.9)	5.6 (0.8)	6.0 (0.6)	5.7 (0.6)	6.0 (0.4)	6.3 (0.9)
**Plasma potassium,** mmol/l	3.9 (0.5)	4.0 (0.5)	4.0 (0.5)	4.0 (0.5)	3.9 (0.6)	4.0 (0.5)	4.0 (0.5)	4.0 (0.5)	4.0 (0.3)	4.0 (0.6)	4.0 (0.3)	4.2 (0.2)
Data presented as mean (standard deviation).												

© 2024 Atila C et al. *JAMA Network Open*.

eFigure 4. Study Flow Diagram

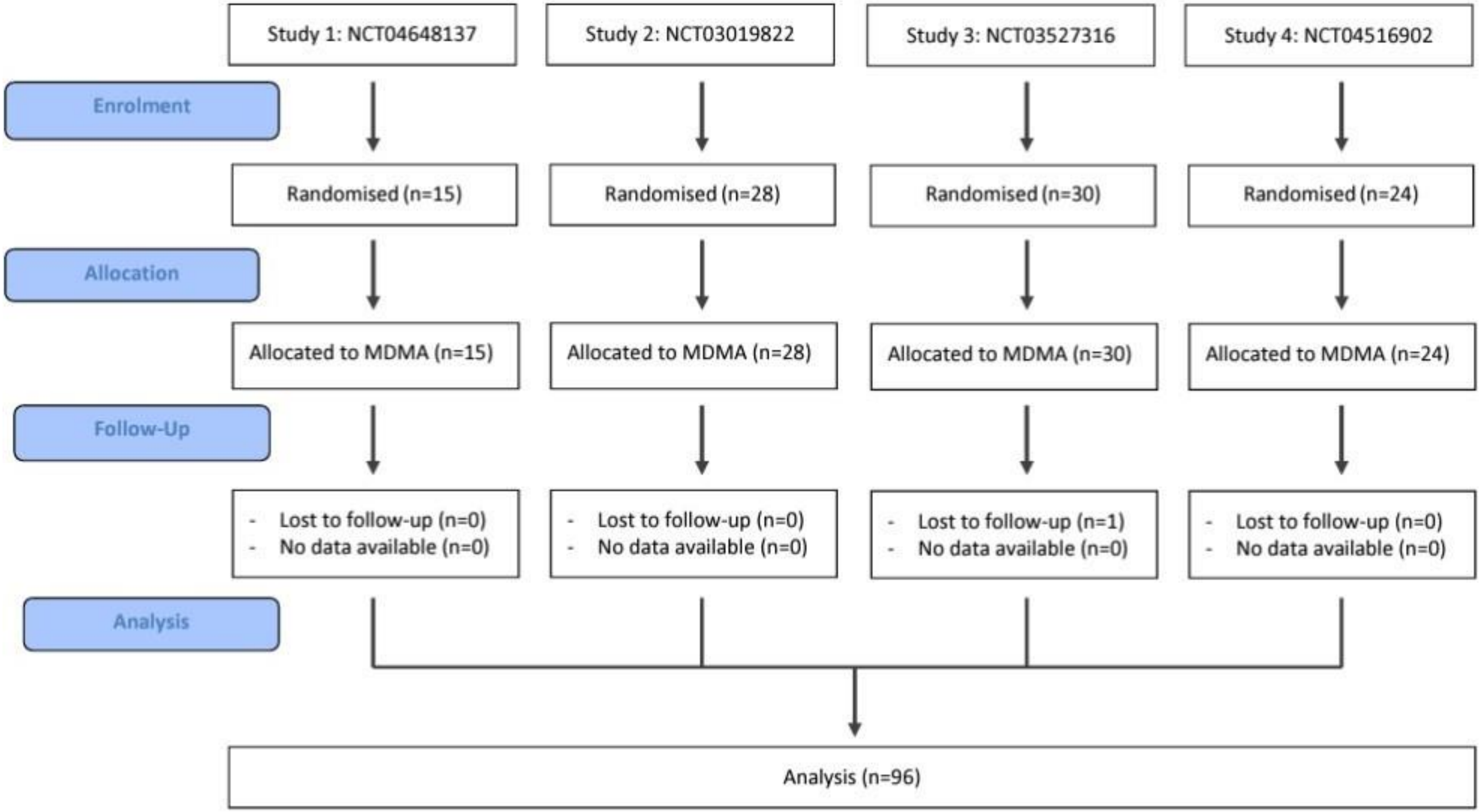

© 2024 Atila C et al. *JAMA Network Open*.
