# Supplementary material for: Oxytocin and the Role of Fluid Restriction in MDMA-Induced Hyponatremia: A Secondary Analysis of 4 Randomized Clinical Trials
Source: JAMA Netw Open. 2024 Nov 15;7(11):e2445278. doi: 10.1001/jamanetworkopen.2024.45278 (PMC11568463; doi:10.1001/jamanetworkopen.2024.45278)
Supplement: Supplement 2. — Trial Protocols [file jamanetwopen-e2445278-s002.pdf]

Circulating oxytocin changes in response to the oxytocin system stimulator MDMA in patients with diabetes insipidus and healthy controls – the OxyMA Study

Study Type:	Other Clinical Trial according to ClinO, Chapter 4
Risk Categorization:	Risk category B, according to ClinO, Art. 61
Study Registration:	clinicaltrial.gov
Sponsor:	Prof. Dr med. Mirjam Christ-Crain<br>University Hospital Basel<br>Endocrinology, Diabetes and Metabolism<br>Petersgraben 4, CH-4031 Basel<br>Phone: +41 61 265 25 25<br>E-Mail: Mirjam.Christ-Crain@usb.ch
Principal Investigator:	Prof. Dr med. Mirjam Christ-Crain
Investigated Intervention:	3,4-methylenedioxymethamphetamine (MDMA)
Project ID:	to be defined
Version and Date:	Version 2.0 (08/10/2020)<br>Version 1.0 (28/08/2020)

CONFIDENTIALITY STATEMENT

The information contained in this document is confidential and the property of the sponsor. The information may not - in full or in part - be transmitted, reproduced, published, or disclosed to others than the applicable Competent Ethics Committee and Regulatory Authority without prior written authorization from the sponsor except to the extent necessary to obtain informed consent from those who will participate in the study.

PROTOCOL SIGNATURE FORM

Study Title	Circulating oxytocin changes in response to the oxytocin system stimulator MDMA in patients with diabetes insipidus and healthy controls – the OxyMA Study
Study ID	To be defined

The Sponsor-Investigator has approved the protocol version 2.0 (dated 08/10/2020) and confirms hereby to conduct the study according to the protocol, current version of the World Medical Association Declaration of Helsinki, and ICH-GCP guidelines as well as the local legally applicable requirements.

Sponsor-Investigator and Principle Investigator:

Name: Prof. Mirjam Christ-Crain

Date: 9.10.20 Signature: M. Christ-Crain

Investigator:

Name: Prof. Dr med. Matthias Liechti

Date: 12.10.20 Signature: [illegible]

Investigator:

Name: Dr. med. Bettina Winzeler

Date: 12.10.20 Signature: [illegible]

Investigator:

Name: Cihan Atila

Date: 09.10.2020 Signature: [illegible]

GLOSSARY OF ABBREVIATIONS **3**
2 BACKGROUND AND RATIONALE **10**
3 STUDY OBJECTIVES AND DESIGN **12**
3.1. Hypothesis and primary objective 12
3.2. Primary and secondary endpoints 12
3.3. Study design 13
3.3.1. Methods of minimizing bias 13
3.4. Study intervention 13
3.5. Study duration 14
4 STUDY POPULATION AND STUDY PROCEDURES **15**
4.1. Inclusion and exclusion criteria, justification of study population 15
4.2. Recruitment, screening and informed consent procedure 16
4.2.1. Recruitment 16
4.2.2. Screening 17
4.2.3. Informed consent procedure 17
4.3. Study procedures 18
4.3.1. Screening visit 18
4.3.2. Main visit (MDMA or placebo) 18
4.3.3. Telephone interview 23
4.4. Withdrawal and discontinuation 26
5 STATISTICS AND METHODOLOGY **27**
5.1. Statistical analysis plan and sample size calculation 27
5.2. Handling of missing data and drop-outs 27
6 REGULATORY ASPECTS AND SAFETY **28**
6.1. Local regulations / Declaration of Helsinki 28
6.2. (Serious) Adverse Events 28
6.3. (Periodic) safety reporting 29
6.4. Radiation 29
6.5. Pregnancy 29
6.6. Amendments 29
6.7. (Premature) termination of study 30
6.8. Insurance 30
7 FURTHER ASPECTS **31**
7.1. Overall ethical considerations 31
7.2. Risk-benefit assessment 31
8 QUALITY CONTROL AND DATA PROTECTION **34**
8.1. Quality measures 34
8.2. Data recording and source data 34
8.3. Confidentiality and coding 34
8.4. Retention and destruction of study data and biological material 35
9 MONITORING AND REGISTRATION **35**
9.1. Monitoring 35
9.2. Registration 35
10 FUNDING / PUBLICATION / DECLARATION OF INTEREST **35**
10.1. Budget and Funding 35
10.2. Publications 36
10.3. DECLARATION OF INTEREST 36
10 REFERENCES **36**

GLOSSARY OF ABBREVIATIONS

AE	*Adverse Event*
ASR/DSUR	*Annual Safety Repot / Development Safety Report*
BASEC	*Business Administration System for Ethical Committees*
CRF	*Case Report Form*
CTCAE	*Common Terminology Criteria for Adverse Events*
FADP	*Federal Act on Data Protection (in German: DSG, in French: LPD, in Italian: LPD)*
eCRF	*electronic Case Report Form*
FOPH	*Federal Office of Public Health*
GCP	*Good Clinical Practice*
HRA	*Human Research Act (in German: HFG, in French: LRH, in Italian: LRUm)*
ICH	*International Conference on Harmonisation*
ClinO	*Ordinance on Clinical Trials in Human Research (in German: KlinV, in French: OClin, in Italian: OSRUm)*
SAE	*Serious Adverse Event*
OT	*Oxytocin*
MDMA	*3,4-methylenedioxymethamphetamine*
GMP	*Good manufacturing practice*
IMP	*Investigational Medical Product*
PI	*Principle Investigator*
T1/2	*Half-time*
Qol	*Quality of Life*
ELISA	*Enzyme-linked Immunosorbent Assay*
RIA	*Radioimmunoassay*
ACTH	*Adrenocorticotropic hormone*
FERT	*Face emotion recognition task*
MET	*Multifaceted empathy task*
STAI	*State-Trait Anxiety Inventory*
TAS-20	*Toronto-Alexithymia-Scale 20*
BDI	*Beck's depression inventory*
SF-36	*Short form health survey*
LC	*List of complaints*
VAS	*Visual analog scale*
CTU	*Clinical Trial Unit*
DKF	*Department für klinische Forschung*
ASZ	*Ambulantes Studienzentrum*
USB	*Unispital Basel*
SOP	*Standard Operating Procedure*
BMI	*Body Mass Index*
SSRI	*Selective Serotonin Reuptake Inhibitor*
MAO	*Monoamine oxidase*
CKD	*Chronic Kidney Disease*
ALAT	*Alanine aminotransferase*
ASAT	*Aspartate aminotransferase*
GGT	*Gamma-glutamyltransferase*
TSH	*Thyroid-stimulating hormone*
LH	*Luteinizing hormone*
FSH	*Follicle-stimulating hormone*
IGF1	*Insulin-like growth factor 1*
fT4	*Thyroxine*

eGFR	*Estimated glomerular filtration rate*
ECG	*Electrocardiography*
DSM-IV	*Diagnostic and Statistical Manual of Mental Disorders, Fourth Edition*
HCT	*Hydrocortisone*
THC	*Tetrahydrocannabinol*
WPW	*Wolff-Parkinson-White*
MedDRA	*Medical Dictionary for Regulatory Activities*

1 STUDY SYNOPSIS

**Sponsor / Sponsor-Investigator**	Prof. Dr. med. Mirjam Christ-Crain, University Hospital Basel
**Study Title**	Circulating oxytocin changes in response to the oxytocin system stimulator MDMA in patients with diabetes insipidus and healthy controls
**Short Title / Study ID**	OxyMA Study
**Protocol Version and Date**	Version 2.0 (dated 08/10/2020)
**Study Registration**	Clinicaltrials.gov (has to be completed)
**Study Category and Rationale**	Physiological interventional study (risk category B, no IMP). The aim of the present study is to investigate the physiological mechanism of inducing oxytocin release using MDMA as a physiological tool and not to use MDMA as a medication or to develop it into a medication (übriger klinischer Versuch Kat. B).
**Background and Rationale**	Disruption of the hypothalamic-pituitary axis due to congenital abnormalities, tumors or head trauma may cause anterior and/or posterior pituitary deficiency also known as partial or panhypopituitarism. Patients with hypopituitarism, especially those with panhypopituitarism (i.e., anterior and posterior insufficiency) often report residual symptoms and lower quality of life despite adequate substitution treatment of deficient pituitary hormones. A recent study identified a potential oxytocin deficient state in men with combined anterior and posterior deficiency. Due to the close proximity of vasopressin and oxytocin, disruption of the vasopressin system leading to diabetes insipidus could as well disturb the oxytocin system leading to low oxytocin levels. It is therefore possible that the increased psychopathology and reduced quality of life as observed in patients with central diabetes insipidus is caused by an oxytocin deficiency.<br>Oxytocin is secreted in a pulsatile manner, is influenced by sex hormones, and socioeconomic functioning. Therefore, measurements of single basal oxytocin levels are insufficient in identifying an oxytocin deficiency. Furthermore, oxytocin measurement is technically cumbersome, and methods are controversial. For other pituitary hormones, e.g. growth hormone or vasopressin, a provocation test to stimulate the respective hormone is often used in case of a suspected hormone deficiency. However, as of today, no provocation test for oxytocin is available and commonly used pituitary provocation tests, such as the hypertonic saline or arginine infusion test, or the oral macimorelin test, do not increase oxytocin levels.<br>3,4-methylenedioxymethamphetamine (MDMA, ecstasy) is used recreationally and to assist psychotherapy for patients with posttraumatic stress disorder. Several studies documented marked acute increases in circulating oxytocin levels in response to MDMA administration as compared to placebo in healthy volunteers.<br>MDMA could therefore be useful as a provocation test to detect an oxytocin deficiency in patients with central diabetes insipidus. We hypothesize that oxytocin provocation following a single dose administration of MDMA is reduced in patients with central diabetes insipidus as compared to healthy volunteers.<br>Our data could improve the pathophysiological understanding of oxytocin in patients with central diabetes insipidus.
**Risk / Benefit Assessment**	**Risk:** Single dose administration of MDMA has been studied frequently and proven to be safe in healthy subjects. The main expected acute adverse effects are consistent with moderate sympathomimetic toxicity including moderate hypertension, tachycardia and a raise in body temperature. Acute psychological distress was observed to be minimal. A slight depressed mood lasting up to three days after MDMA application has been observed. The patients will be monitored closely during MDMA application and until the acute effects wear off. Participants will receive a phone call 3 days after the intervention to asses mood changes.<br><br>**Benefit:** We do not expect immediate benefit to the study participants. Our data could provide a potential provocation test for oxytocin and clarify whether patients with diabetes insipidus have an oxytocin deficient state.
**Objectives**	The primary objective of this study is to evaluate oxytocin levels in response to MDMA administration as compared to placebo in patients with diabetes insipidus and healthy volunteers.

	Secondary objectives are to evaluate physical and emotional changes between MDMA and placebo for patients with central diabetes insipidus and healthy volunteers:<br>A. Evaluation of maximum OT peak following MDMA<br>B. Time course of plasma OT levels<br>C. Time course of plasma concentrations of MDMA<br>D. Changes of other endocrine hormones<br>E. Change of subjective feelings<br>F. Change of mood perception<br>G. Change of empathy<br>H. Change in anxiety levels<br>F. Change in alexithymia<br>G. Change in depressed mood<br>H. Change in general health status<br>I. Safety measurements
**Endpoints**	The primary endpoint is the area under the concentration time curve in oxytocin level from baseline oxytocin measurement (before intake) to 6 hours after a single administration of MDMA (100mg) as compared to placebo in the same subjects between patients with central diabetes insipidus and healthy volunteers.<br><br>Secondary outcomes will be assessed between MDMA as compared to placebo for patients with central diabetes insipidus and healthy volunteers:<br>A. Peak change in OT plasma level<br>B. Time course of plasma OT levels<br>C. Time course of plasma MDMA concentration<br>D. Time course of cortisol, prolactin, copeptin and ACTH levels<br>E. Subjective/emotional effects assessed on a 10-point visual analog scale (e.g., feelings of anxiety, pleasure, fear, 0-10)<br>F. Recognition of negative emotions in the face emotion recognition task (FERT)<br>G. Empathy in the multifaceted empathy task (MET)<br>H. Anxiety level with the State-Trait Anxiety Inventory (STAI)<br>F. Level of Alexithymia using the Toronto-Alexithymia-Scale 20 (TAS-20)<br>G. Level of depression using the Beck-Depressions-Inventory II (BDI-II)<br>H. Level of general physical & mental health using the short form health survey (SF-36)<br>I. Safety: Assessments of clinical/laboratory variables (blood pressure, heart rate, body temperature, plasma sodium)
**Study Design**	Randomized, double-blind, placebo-controlled, cross-over (MDMA versus placebo, within-subject comparison) study in patients with central diabetes insipidus versus healthy controls (between-subject comparison)
**Statistical Considerations**	**Hypothesis**<br>The primary endpoint is the area under the concentration time curve in oxytocin level from baseline oxytocin measurement (before intake) to 6 hours after a single administration of MDMA (100mg) as compared to placebo in the same subjects between patients with central diabetes insipidus and healthy volunteers.<br><br>The null hypothesis is that the area under the concentration time of oxytocin is equal for patients with central diabetes insipidus and healthy volunteers after a single dose of MDMA, i.e. the primary endpoint is zero. The alternative hypothesis is that the area under the concentration time of oxytocin is lower in patients with central diabetes insipidus as compared to healthy volunteers, i.e. the primary endpoint is not zero.<br><br>**Statistical Analysis**<br>The primary endpoint, area under the concentration time curve for oxytocin will be analyzed for a difference from zero for patients with central diabetes insipidus and healthy volunteers. The p-value from the test will be reported.<br>The time course of oxytocin measures after intake of MDMA and placebo will be visualized by means of line plots and boxplots for patients with central diabetes insipidus and healthy volunteers. Summary statistics (median, interquartile range, minimum and maximum value) will be presented for each measurement time of oxytocin for MDMA and placebo for patients with central diabetes insipidus and healthy volunteers. Maximum oxytocin time will be

	summarized for MDMA and placebo for patients with central diabetes insipidus and healthy volunteers separately by means of frequency distribution according to the measurement times, and by median, interquartile range, minimum and maximum value.<br>Oxytocin area under the concentration time curve from baseline to 6 hours after intervention will be tested for a difference between MDMA and placebo for patients with central diabetes insipidus and healthy volunteers. Summary statistics (median, interquartile range, minimum and maximum value) will be presented separately.<br>All other secondary endpoints will be analyzed descriptively. The time course of MDMA, cortisol, prolactin, copeptin, and ACTH levels will be visualized by means of line plots and boxplots, and summary statistics will be presented for each measurement time for MDMA and placebo for patients with central diabetes insipidus and healthy volunteers separately.
**Inclusion- / Exclusion Criteria**	**Inclusion criteria diabetes insipidus**:<br>1. Confirmed diagnosis of central diabetes insipidus<br>2. Age 18 - 65 years<br><br>**Inclusion criteria healthy volunteers**:<br>1. Healthy volunteers<br>2. Matched for age, sex, BMI and estrogen replacement/menopause/hormonal contraceptives to patients with central diabetes insipidus<br>3. No medication, except hormonal contraception<br><br>**Exclusion criteria**:<br>1. Familial central diabetes insipidus<br>2. Participation in a trial with investigational drugs within 30 days<br>3. Illicit substance use (with the exception of cannabis) more than 10 times in lifetime or any time within the previous two months<br>4. Consumption of alcoholic beverages >15 drinks/week<br>5. Tobacco smoking >10 cigarettes/day<br>6. Cardiovascular disease (coronary artery disease, heart failure LVEF <40%, stroke in the last 3 months, atrial fibrillation/flatter, WPW-Syndrome)<br>7. Uncontrolled arterial hypertension (>140/90 mmHg) or hypotension (syst blood pressure <85mmHg)<br>8. Current or previous major psychiatric disorder (e.g., major depression, schizophrenia spectrum disorder)<br>9. Psychotic disorder in first-degree relatives<br>10. Regular intake of SSRI, MAO-Inhibitors<br>11. Pregnancy and breastfeeding<br>12. Diagnosed CKD > grade III (GRF < 30ml/min)<br>13. Diagnosed liver cirrhosis or alanine aminotransferase (ALAT) or aspartate aminotransferase (ASAT) levels 2.5 times above the normal range
**Number of Participants with Rationale**	**15 patients with primary polydipsia and 15 healthy volunteers**<br>**Determination of sample size**<br>Sample size was estimated in order to show a significant increase in oxytocin after the intake of a single oral dose of MDMA with a power of 90 % and a significance level α of 0.05.<br>Sample size estimation was based on preliminary data from 28 healthy adults. In this trial, oxytocin levels following MDMA intake was studied. The mean increase in oxytocin levels for MDMA was 809+/-64 pg/mL and 259 +/- 62 pmol for placebo.<br>Assuming 30% reduced levels of oxytocin after MDMA in patients with diabetes insipidus as compared to healthy volunteers, a total of 15 subjects should be recruited in order to have N = 12 evaluable subjects, assuming a drop-out rate of 20 %.
**Study Intervention**	3,4-methylenedioxymethamphetamine (MDMA, ecstasy) will be prepared as gelatin capsules with mannitol as the filler. MDMA will be administered in a single absolute dose of 100 mg corresponding to a medium high dose of (mean ± SD) 1.3 ± 0.3 mg/kg body weight
**Control Intervention**	Identical placebo (only mannitol) capsules will be prepared.
**Study procedures**	Study participants will present at three separate days at the outpatient study center, Departement Klinische Forschung (DKF), University of Basel.<br><br>**Screening visit:** Participants will be informed about all procedures involved in the study. A history, physical examination and blood screening will be performed to evaluate eligibility. If included in the study, participants will be randomized to receive either first placebo or first

	MDMA, respectively. Participants, study investigators and study nurses will be blinded to the study intervention.<br><br>**Main visit:** During the main visits, participants will present in the morning at the study site. An indwelling intravenous catheter will be placed in an antecubital vein for blood sampling. MDMA or placebo will be administered one hours later. Autonomic and subjective/emotional effects will be assessed repeatedly throughout the session (timepoint 0 min, 30min, 60 min, 90 min, 120 min, 180min, 240 min, 300 min, 360 min). Blood will be collected to determine oxytocin, MDMA and plasma sodium levels at 0 min, 90 min, 120 min, 150min, 180min and 300 min. Cortisol, prolactin, copeptin and ACTH will be taken at 0 min, 90 min, 150min and 300 min. Approximately 2-2.5 hours after MDMA or placebo administration during the expected peak concentration participants will perform the following tasks: Face emotion recognition (FERT) and multifaceted empathy task (MET). A standardized list of complaints and the State-Trait Anxiety Inventory (STAI-S) will be performed on baseline (timepoint 0min) and repeated at timepoint 180min (STAI-S), timepoint 360min (LC). Subjects will be under continuous medical supervision until any alterations of consciousness have completely subsided, this is expected within 6h.<br><br>After a washout-phase of at least 2 weeks, visit 2 will be scheduled. At Visit 2, participants will undergo the same procedure as described above with the second intervention. In female participants, the main visits will preferentially be planned during the mid-follicular phase or if on estrogen replacement therapy, during the active treatment phase.<br><br>**Telephone interview:** 3 days after the main visits, participants will receive a phone call to inquire for subjective feeling and adverse events.
**Study Duration and Schedule**	Estimated duration for the main investigational plan, i.e. from start of screening of first participant to last participant last visit and finishing the study will take approximately 4-5 weeks. The total duration per participants will be 18h.<br><br>Planned 10/2020 of First-Participant-In<br>Planned 10/2021 of Last-Participant-Out
**Senior Investigators**	Prof. Dr. med. Mirjam Christ-Crain<br>Division of Endocrinology, Diabetes and Metabolism<br>University Hospital Basel, Petersgraben 4, 4031 Basel, Switzerland<br><br>Prof. Dr. med. Matthias Liechti<br>Division of Clinical Pharmacology and Toxocology<br>University Hospital Basel, Petersgraben 4, 4031 Basel, Switzerland
**Study Center**	Single-centre study<br>Endocrinology, Diabetes and Metabolism<br>University Hospital Basel, Switzerland
**Data privacy**	The investigator affirms and upholds the principle of the participant's right to privacy and that they shall comply with applicable privacy laws. Especially, anonymity of the participants is guaranteed when presenting the data at scientific meetings or publishing them in scientific journals.<br>Individual patient medical information obtained as a result of this study is considered confidential and disclosure to third parties is prohibited. Patient confidentiality will be further ensured by utilizing patient identification code numbers to correspond to treatment data in the computer files.<br>For data verification purposes, authorized representatives of the Sponsor or EKNZ may require direct access to parts of the medical records relevant to the study, including participants' medical history.
**Ethical consideration**	This study will identify the role of oxytocin in patients with central diabetes insipidus. Recent findings have suggested low oxytocin levels in relation to increased psychopathology in patients with central diabetes insipidus. However, oxytocin measurements are difficult, and no provocation test is yet available. This study will improve the pathophysiological implications of oxytocin in patients with central diabetes insipidus by clarifying whether these patients indeed have oxytocin deficiency, which is only possible to show by using a provocation test, here

	MDMA. Future studies could consider interventional studies using oxytocin application to reduce pathophysiology in patients with central diabetes insipidus and ultimately increase their quality of life.<br>The cross-over design with a washout period of at least two weeks is considered appropriate as first, no residual effects of MDMA is present after two weeks, second, the sample size is reduced as the inter-individual variability is reduced.<br>In order to increase the understanding of the pathophysiology of oxytocin and MDMA in patients with central diabetes insipidus it is crucial to compare the values to healthy controls. Only with these two study populations can we conclude how oxytocin is stimulated following MDMA.
**GCP Statement**	This study will be conducted in compliance with the protocol, the current version of the Declaration of Helsinki, the ICH-GCP, the HRA as well as other locally relevant legal and regulatory requirements.

2 BACKGROUND AND RATIONALE

Disruption of the hypothalamic-pituitary axis due to congenital abnormalities, tumors or head trauma may cause anterior and/or posterior pituitary deficiency also known as partial or panhypopituitarism. Patients with hypopituitarism, especially those with panhypopituitarism (i.e., anterior and posterior deficiency) often report residual symptoms and lower quality of life (QoL) despite adequate substitution treatment of deficient pituitary hormones and often persists over several years of treatment and still does not reach population norms [1- 5].

The neuropeptide oxytocin (OT) is produced in the paraventricular nucleus and supraoptic nucleus of the hypothalamus and released into the circulation from the axon terminals that project to the posterior pituitary gland. OT is secreted in a pulsatile manner [6,7] is influenced by sex hormones, and socioeconomic functioning. It plays an important role during labour inducing uterus contraction, lactation and psychosocial functions, including attachment, pair bonding, socioemotional processing and parenting [8–15].

Due to the close proximity of vasopressin and OT, disruption of the vasopressin system leading to central diabetes insipidus could as well disturb the OT system leading to low OT levels. It is therefore possible that the increased psychopathology and reduced QoL as observed in patients with central diabetes insipidus is caused by an OT deficiency [16]. Accordingly, recent studies identified lower OT levels in men with combined anterior and posterior pituitary deficiency [17,18] compared to healthy controls, however, another recent study did not confirm these findings [19]. This controversy might also be due to difficulties in measurement of OT. Measurements of single basal OT levels are insufficient in identifying an OT deficiency [20, 21] Furthermore, OT measurement is technically cumbersome, and methods as well as the ideal sampling are controversial [20, 21]. Published studies used different samples e.g. blood [17, 19], salivia [9, 22, 23], cerebrospinal fluid [24] and urine [25], different laboratory methods e.g. bioassays [26] such as ELISA [19] or RIA [17] and different measurement methods e.g. single basal level [19] or pooled blood analysis [17]. For other pituitary hormones, e.g. growth hormone or vasopressin, a provocation test to stimulate the respective hormone is often used in case of a suspected hormone deficiency. Yet, no standard provocation test for OT measurement is established [20, 21], commonly used pituitary provocation tests, such as the hypertonic saline or arginine infusion test, or the oral macimorelin test, do not increase OT levels [27, 28] [own unpublished data].

3,4-methylenedioxymethamphetamine (MDMA, “ecstasy”) is used recreationally and to assist psychotherapy for patients with posttraumatic stress disorder [29–32]. Effects of MDMA showed many similarities to OT on emotion processing and social interaction, such as increased trust, closeness to others, identification of facial emotions, openness and productive communication

[33–36]. Several studies documented marked increases in circulating OT levels in response to MDMA administration as compared to placebo in healthy volunteers [25, 37–42]. However, no data in patients with central diabetes insipidus, where a potential OT deficiency has been described, exist. MDMA could therefore be useful to improve the pathophysiological understanding of OT in patients with central diabetes insipidus.

3 STUDY OBJECTIVES AND DESIGN

3.1. Hypothesis and primary objective

We hypothesize that OT provocation following a single dose administration of MDMA is reduced in patients with central diabetes insipidus as compared to healthy volunteers. The primary objective of this study is to evaluate OT levels in response to MDMA administration as compared to placebo in patients with central diabetes insipidus and healthy volunteers.

Secondary objectives are to evaluate physical and emotional changes between MDMA and placebo for patients with central diabetes insipidus and healthy volunteers:

A. Evaluation of maximum OT peak following MDMA
B. Time course of plasma OT levels
C. Time course of plasma concentrations of MDMA
D. Changes of other endocrine hormones
E. Change of subjective feelings
F. Change of mood perception
G. Change of empathy
H. Change in anxiety levels
F. Change in alexithymia
G. Change in depressed mood
H. Change in general health status
I. Safety measurements

3.2. Primary and secondary endpoints

The primary endpoint is the area under the concentration time curve in OT level from baseline OT measurement (before intake) to 6 hours after a single administration of MDMA (100mg) as compared to placebo in the same subjects between patients with central diabetes insipidus and healthy volunteers.

Secondary outcomes will be assessed between MDMA as compared to placebo for patients with central diabetes insipidus and healthy volunteers:

A. Peak change in OT plasma level
B. Time course of plasma OT levels
C. Time course of plasma MDMA concentration
D. Time course of cortisol, prolactin, copeptin and ACTH levels
E. Subjective/emotional effects assessed on a 10-point visual analog scale (e.g., feelings of anxiety, pleasure, fear)
F. Recognition of negative emotions in the face emotion recognition task (FERT)

G. Empathy in the multifaceted empathy task (MET)

H. Anxiety level with the State-Trait Anxiety Inventory (STAI)

F. Level of Alexithymia using the Toronto-Alexithymia-Scale 20 (TAS-20)

G. Level of depression using the Beck-Depressions-Inventory II (BDI-II)

H. Level of general physical & mental health using the short form health survey (SF-36)

I. Safety: Assessments of clinical/laboratory variables (blood pressure, heart rate, body temperature, plasma sodium)

3.3. Study design

This is a randomized, double-blind, placebo-controlled, cross-over (MDMA versus placebo, within-subject comparison) study in patients with central diabetes insipidus versus healthy controls (between-subject comparison).

3.3.1. Methods of minimizing bias

Randomization

After successful screening, participants will be randomized to be given MDMA or placebo first in random order, using block randomization to counterbalance for the order of MDMA or placebo and the groups (patients or healthy subjects). Each subject will participate in two main visits. The GMP facility (Apotheke Dr. Hysek, Biel, Switzerland) will perform the randomization. The intervention order is counterbalanced. An intervention order is assigned to each subject number (code list) and kept by the GMP facility. Only the GMP facility and the PI have access to the code (sealed envelopes).

Blinding procedures

The order of the two interventions is randomly predefined in a randomization list. The randomization list is not known to the patients, the investigators and the study nurses involved in the trial. Subjects and study personnel involved in supervising the session will be blinded to treatment order. The order is balanced.

Unblinding Procedures (Code break)

Only the GMP facility and the PI have access to the sealed code. In case of a medical emergency and in case of premature study termination the code can be accessed by the PI or the pharmacist of the GMP facility.

3.4. Study intervention

MDMA will be prepared as gelatin capsules containing 25 mg of pharmaceutically pure MDMA hydrochloride (ReseaChem GmbH, Burgdorf, Switzerland) and mannitol filler. Placebo will be prepared as identical gelatin capsules containing only mannitol filler. All products are prepared

by a Swissmedic-approved GMP facility (Apotheke Dr. Hysek, Biel, Switzerland) according to GMP guidelines and with the authorization of the BAG.
MDMA will be administered in a single dose of 100 mg (4 capsules of 25 mg MDMA). The MDMA dose was chosen based on previous studies using oral administered MDMA with dosages between 75mg and 125mg [43–46]. In controlled clinical [30-47] setting MDMA is considered to be safe with moderate subjective and cardiovascular effects [44, 48]. The peak effects of MDMA are expected after 2 h [44, 48–50]. $T_{1/2}$ of MDMA (100 -125 mg dose) is about 8-10 h [44, 51]. Effects of MDMA last about for 6 h [44].

Substance accountability and storage

The study substances are stored in a locked and temperature-controlled drug cabinet at the Ambulatory Study Center/Department of Endocrinology. A drug dispensing and accountability log for the study substances will be kept current. The person dispensing the substances registers and signs out the dispensing date, participant number, and treatment sequence.

3.5. Study duration

Estimated duration for the main investigational plan, i.e. from start of screening of first participant to last participant last visit and finishing the study will take approximately 4-5 weeks. The total duration per participants will be 18 hours; one screening visit of 2 hours, two main study visits of 7,5 hours and two telephone interviews of 20 minutes each.

Planned 10/2020 of First-Participant-In
Planned 10/2021 of Last-Participant-Out

4 STUDY POPULATION AND STUDY PROCEDURES

4.1. Inclusion and exclusion criteria, justification of study population

Inclusion criteria diabetes insipidus:

1. Confirmed diagnosis of central diabetes insipidus
2. Age 18 - 65 years

Inclusion criteria healthy volunteers:

1. Healthy volunteers
2. Matched for age, sex, BMI and estrogen replacement/menopause/hormonal contraceptives to patients with central diabetes insipidus
3. No medication, except hormonal contraception

Exclusion criteria:

1. Familial central diabetes insipidus
2. Participation in a trial with investigational drugs within 30 days
3. Illicit substance use (with the exception of cannabis) more than 10 times in lifetime or any time within the previous two months
4. Consumption of alcoholic beverages >15 drinks/week
5. Tobacco smoking >10 cigarettes/day
6. Cardiovascular disease (coronary artery disease, heart failure LVEF <40%, stroke in the last 3 months, atrial fibrillation/flatter, WPW-Syndrome)
7. Uncontrolled arterial hypertension (>140/90 mmHg) or hypotension (syst blood pressure <85mmHg)
8. Current or previous major psychiatric disorder (e.g., major depression, schizophrenia spectrum disorder)
9. Psychotic disorder in first-degree relatives
10. Regular intake of SSRI, MAO-Inhibitors
11. Pregnancy and breastfeeding
12. Diagnosed CKD > grade III (GRF < 30ml/min)
13. Diagnosed liver cirrhosis or alanine aminotransferase (ALAT) or aspartate aminotransferase (ASAT) levels 2.5 times above the normal range

Justification of study population:

Adult patients suffering from central diabetes insipidus will participate in the study after thorough information and providing written informed consent. This study population has been chosen because of a potential OT deficiency and the possible relation to striking psychopathology in these patients [17, 18]. Several studies showed a lower QoL in patients with central diabetes insipidus

despite adequate hormone substitution [52]. Lack of knowledge regarding a possible relation between lower QoL and OT leads to investigate OT levels in patients with central diabetes insipidus. Many common provocation tests used for detecting hormonal deficiency, e.g., hypertonic saline or arginine infusion test and oral macimorelin, showed no increase in OT response in healthy controls and are therefore not useful to investigate a potential OT deficiency in patients with central diabetes insipidus [27] [own unpublished data].

The OT system stimulator MDMA has shown in many studies a remarkable increase in OT blood levels in healthy volunteers. MDMA used in experimental studies in humans indicate that the likelihood for significant toxicity from doses of MDMA such as the one to be used in the present study (i.e. 100mg) and in controlled settings is very low. MDMA is in general well tolerated and common side effects (see section 7.2.) are describes as moderate and tolerable [43]. Non-specifically desired side effects for this study include mainly an increase in sympathomimetic tone including an increase in blood pressure and heart rate and positive subjective mood effects [43]. Long-term effects have been reported with extensive use of ecstasy but not with moderate exposure and rarely with one-time usage of single moderate doses of MDMA [35, 52]. We therefor do not expect lasting biological or psychological injury for patients with diabetes insipidus and healthy participants. At the University Hospital of Basel, Department of Clinical Pharmacology, MDMA has been used in approximately 200 subjects and over 300 experimental sessions [38, 41, 42, 53, 54]. There were no serious adverse reactions.

4.2. Recruitment, screening and informed consent procedure

4.2.1. Recruitment

Patients with central diabetes insipidus will be mainly recruited at the Department of Endocrinology, University Hospital Basel. All patients with central diabetes insipidus need a previously established diagnosis e.g. documented water deprivation test, hypertonic saline or arginine stimulation test.

If an eligible patient is seen by a treating endocrinologist, he or she will inform the study physician about the patient /patients with central diabetes insipidus presenting for a routine follow-up at the outpatient clinic will be asked to participate the study by the Endocrinologist. Additionally, the electronic outpatient record system will be used to prescreen patients with central diabetes insipidus and will be contacted either through the treating endocrinologist or the study physician. Patients and healthy subjects will be recruited by an advertisement on the webpage of the University of Basel. After a brief information by telephone, the participants will receive the ''Patienten- und Probandeninformation'' and the ''Einverständniserklärung'' by mail or by e-mail. After that, patients willing to participate in the study can contact the investigators, ask questions and arrange the screening visit.

4.2.2. Screening

The subjects will be informed about the study both verbally and by the approved written consent form regarding the altered state of consciousness, the study procedures and associated risks; they will be requested to sign the informed consent. Subjects will be examined by the study physician. Basic health will be ensured by general medical examination including medical history, physical examination, ECG, standardized blood pressure measurement, and blood chemistry/hematology. Body weight and height will be measured at the screening visit.

Subjects will be screened using a semi-structured clinical interview for DSM-IV to exclude those with a personal or family (first-degree relative) axis I major psychiatric disorder or a history of illicit drug dependence. The psychiatric interview is conducted by a trained study physician who decides whether subjects meet the psychiatric inclusion criteria.

Occasional recreational drug use in the past (<10 times) is not an exclusion criterion if no adverse reactions occurred and if use was moderate and controlled. Subjects will be asked to abstain from any illicit drug use during the study to abstain from excessive alcohol consumption between test-sessions (not more than 15 drinks/week) and in particular not to drink alcohol the day before the test sessions. A routine laboratory blood test is performed at the screening examination including plasma electrolytes, plasma osmolality, ALAT, ASAT, GGT, creatinine, eGFR and blood hematology.

4.2.3. Informed consent procedure

The investigators will explain to each patient with central diabetes insipidus or healthy volunteer the nature of the study, its purpose, the procedures involved, the expected duration, the potential risks and benefits and any discomfort it may entail. Each participant will be informed that the participation in the study is voluntary and that he or she may withdraw from the study at any time and that withdrawal of consent will not affect his or her subsequent medical assistance and treatment. The participant will be informed that his or her medical records may be examined by authorized individuals other than their treating physician.

All participants for the study will be provided a participant information sheet and a consent form describing the study and providing sufficient information for participant to make an informed decision about their participation in the study. Enough time needs to be given to the participant to decide whether to participate or not.

The formal consent of a participant, using the approved consent form, will be obtained before the participant is submitted to any study procedure.

The consent form will be signed and dated by the investigator or his designee at the same time as the participant sign. A copy of the signed informed consent will be given to the study participant. The consent form will be retained as part of the study records. Neither patients nor the healthy participants will have a direct benefit from the study. Therefore, as a compensation for their time,

patients and healthy participants will receive a compensation after the study participation of 400 sFr. Additionally, travel costs for patients not living in the area will be reimbursed.

4.3. Study procedures

Study participants will present at three separate days at the outpatient study center, Ambulatory Study Center (ASZ), University of Basel (USB). As seen in the figure above, if enrolled in the study patients and healthy volunteers will be randomized to receive either the placebo or MDMA first. After a wash-out phase of at least 2 weeks, the second visit of the study will be planned with either MDMA or placebo. The screening procedure, main visit and telephone interview are described in detail in the following paragraphs:

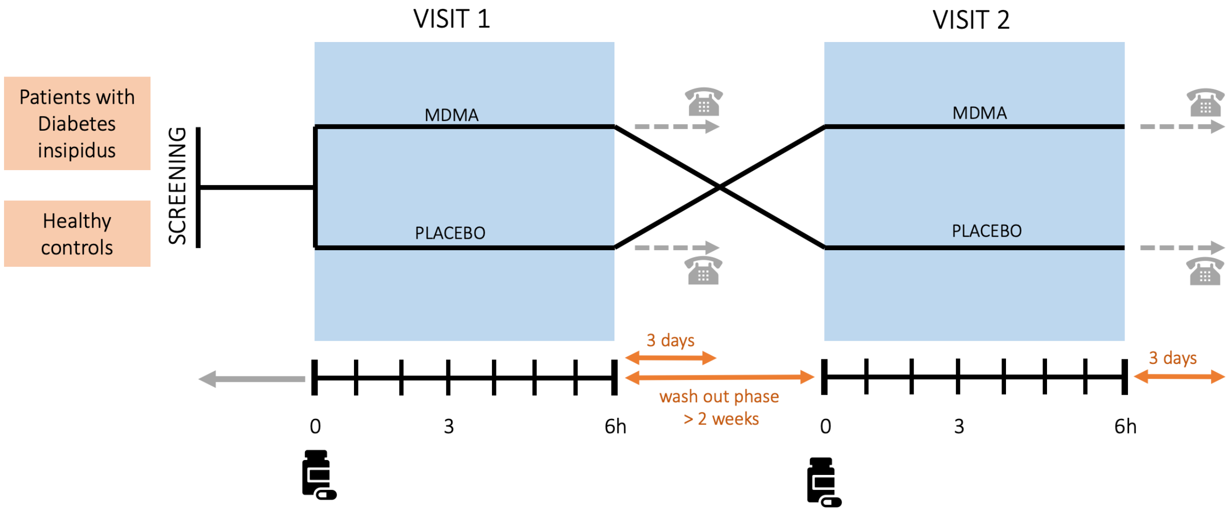

4.3.1. Screening visit

Participants will be informed about all procedures involved in the study. A medical history, physical examination and blood screening will be performed to evaluate eligibility. For detailed information see 4.2.2.

If included in the study, patients or healthy participants will be randomized to receive either first placebo or first MDMA, respectively. Patients or healthy participants, study investigators and study nurses will be blinded to the study intervention. Eligible patients or healthy participants will undergo a detailed psychological baseline evaluation right after the screening at the same day. The psychological baseline evaluation includes the assessment of anxiety level by using the State-Trait Anxiety Inventory (STAI), scoring the mood using the Beck's Depression Inventory (BDI II), the degree of alexithymia using the Toronto Alexithymia Scale (TAS-20) and general health status using the Short Form (36) Health Survey (SF-36). No more than 2 months should be between the screening visit and the main study visit.

4.3.2. Main visit (MDMA or placebo)

During the main visits, participants will present in the morning at the study site. An indwelling intravenous catheter will be placed in an antecubital vein for blood sampling. To avoid clotting participants will receive continuous 0,9% NaCl infusion during the whole visit with a total of maximum of 250 ml (40 ml/h). Patients with central diabetes insipidus and secondary adrenal insufficiency on hydrocortisone (HCT) substitution will be asked to take 50 mg HCT instead of the regular dose for the main visits (30mg morning dose and 20mg noon dose), desmopressin will be taken by the patients as regular.

Pregnancy test /drug screening: The participants will be screened for pregnancy and illicit drugs using a urinary test before starting the session at each visit. Positive screens for stimulants, opioids or tranquilizers will result in exclusion from the study. Positive screens for THC (cannabis) will be recorded but do not result in study exclusion. This is because THC consumption can be detected in urine for up to several weeks and use days before a study day is unlikely to affect the outcome.

Questionnaires: A standardized list of complaints (LC) (see further information below) and the STAI-S will be performed on baseline (timepoint 0min) and repeated at timepoint 180min (STAI-S), timepoint 360min (LC).

Vital parameters: Blood pressure (systolic and diastolic), heart rate and body temperature will be measured at several timepoints during the main visit (timepoint -60 min, 0 min, 30min, 60 min, 90 min, 120 min, 150min, 180min, 210min, 240 min, 270min, 300 min, 330min, 360 min).

Study Drug: MDMA (100mg) or placebo will be administered orally one hours after the start of the main visit.

Subjective effects: Autonomic and subjective/emotional effects will be assessed repeatedly throughout the main visit (timepoint 0 min, 30min, 60 min, 90 min, 120 min, 150min, 180min, 240 min, 300 min, 360 min) using subjective effects questionnaire assessed with visual analog scales (VAS).

Blood samples: Samples will be collected on the study days as shown in Table 2. Blood will be collected to determine OT, MDMA and plasma sodium level at 0 min, 90 min, 120 min, 150 min, 180min and 300 min. Cortisol, prolactin, copeptin and ACTH concentrations will be taken at 0 min, 90 min, 150 min and 300 min. The blood samples will be taken as aliquots for the later batch analysis and will be centrifuged at 4°C at 3000 rpm for 10 min, and then stored at -80°C until analysis. Plasma sodium level will be taken for instant analysis. A basal hormonal status will be

conducted at baseline in main visit 1 at timepoint 0 min including TSH, fT4, IGF1, FSH. LH, Testosterone and Estradiol. Blood concentrations are determined in our laboratory using validated and previously used methods.

Amount of blood sampling: The total amount of blood collected during the study is 310 ml. This amounts to less than a standard blood donation and spread out over a time frame of 8 -10 weeks. The risk is there for acceptable for the participants.

Computer test: At timepoint 150 min during the expected peak concentration of MDMA participants will perform the following tasks: Face emotion recognition (FERT) and multifaceted empathy task (MET). The test will take 25 minutes in total for the participant. See section bellow for further information.

Standardized meal / fluid intake: The subject will be allowed to drink to thirst – the total fluid intake will be (in addition to 250ml i.v.) recorded during the main visit. We will offer a standardized breakfast and lunch at timepoint -60 min and timepoint 180 min.

End of session: Subjects will be under continuous medical supervision until any alterations of consciousness have completely subsided, this is expected within 6h for MDMA. After the test session subjects are allowed to leave only if the subjective effects have ceased (VAS score "drug effect" back to predose ± 1) and as assessed by the investigator. No close monitoring is needed beyond this time and subjects can return home as also was the case in a similar study [39, 43, 55].
Subjects are prohibited to drive a car or to operate any machines within 48 h of substance administration. As for any complications resulting from this clinical study the investigators will provide assistance for treatment and the costs are covered by the study insurance. Any AE between sessions will be recorded at the beginning of the next session or telephone interview. Safety follow-up meetings beyond this time are not needed [57]. However, subjects will be provided with the phone number of the study physician, and the study leader in case they need assistance, or should medical problems arise.

Second main visit: After a washout-phase of at least 2 weeks, visit 2 will be scheduled. At Visit 2, participants will undergo the same procedure as described above with the second intervention. In female participants, the main visits will preferentially be planned during the mid-follicular phase or if on estrogen replacement therapy, during the active treatment phase

Adverse effects and list of complaints (LC)

The LC consists of 66 items, yielding a global score measuring physical and general discomfort [88]. The LC list is administered at the beginning and at the end of the session with reference to complaints throughout the entire session. Subjects will also be asked to report any adverse events during the sessions or between study sessions at the beginning of the next session. The test requires about 2 minutes.

State-Trait Anxiety Inventory (STAI - G) and STAI (STAI- S)

This is a questionnaire given to adults to determine the general anxiety levels. Based on responses to 20 items, with scores ranging from 1 ("almost never") to 4 ("almost always"), a total score is calculated. The total Trait scores range from 20 to 80, with higher scores indicating more pronounced anxiety and scores. A score above 45/80 indicating clinically significant anxiety symptoms. The State Anxiety Scale (S-Anxiety/STAI-S) evaluates the current state of anxiety, asking how respondents feel "right now," using items that measure subjective feelings of apprehension, tension, nervousness, worry, and activation/arousal of the autonomic nervous system. The Trait Anxiety Scale (T-Anxiety/STAI-G) evaluates relatively stable aspects of "anxiety proneness," including general states of calmness, confidence, and security [58]. We will use the STAI-G for the screening and the STAI-S for the main visits. The test requires about 5 minutes.

Short form 36 Health survey (SF 36)

The SF 36 is a self-reported measure of health status with 36 items which assesses eight multi-item variables: Physical functioning (10 items), social functioning (2 items), role limitations due to emotional problems (3 items), mental health (5 items) energy and vitality (4 items), pain (2 items and general perception of health (5 items) [59]. The subjects will be asked to fill out the form at the screening visit. The questionnaire requires about 5 minutes.

Toronto Alexithymia Scale 20 (TAS 20)

Alexithymia is described as a trait to identify and describe emotions experienced by oneself or others. It is characterized by a marked difficulty in consciously experiencing, identifying, and describing emotions, as well as reduced introspection. The TAS 20 has a three-factor structure: difficulty identifying feelings, difficulty describing feelings and externally oriented thinking. It includes 20 questions with scores ranging from 1 (strongly disagree) to 5 (strongly agree) [59, 60]. We will use the TAS 20 as a baseline evaluation at the screening visit. The questionnaire requires about 2 minutes.

Beck's Depression Inventory II (BDI II)

The BDI II is one of the most used self-reported scales for measuring depression. It uses 21 items ranked from 0 (symptom absent) to 3 (severe symptoms) to measure the severity of depression. The self-administered form takes about 5-10 minutes for the participant. The minimum score is 0 and maximum score is 63. In non-clinical populations, scores above 20 indicate depression. In those diagnosed with depression, scores of 0–13 indicate minimal depression, 14–19 (mild depression), 20–28 (moderate depression) and 29–63 (severe depression) [61, 62]. We will use the BDI II as a baseline evaluation of depression at the screening visit. The questionnaire requires about 5 minutes.

Subjective effects questionnaire (Visual Analog Scales, VAS)

VAS will be repeatedly used to assess subjective alterations in consciousness over time. VAS will be presented as a range from 0 to 10 marked with "not at all" on the left and "extremely" on the right [38, 42, 63]. The following VAS will be used: "any effect", "good effect", "bad effect", "liking", "high", "happy", "fear", "stimulated", "feeling close to others", "concentration", "thinking", "open", "trust", "want to be with other people", "loss of sense of time", and "the boundaries between myself and my surroundings seemed to blur". Scales will be administered before and repeatedly after substance administration and will take 2 minutes to answer. The maximal ratings (E_{max}, 0-10) are defined for each VAS and E_{max} values compared between treatments using analysis of variance (ANOVAs).

Facial emotion recognition task (FERT)

The FERT is used to assess recognition of basic emotions. The FERT was sensitive to the effects of other psychoactive substances like MDMA [40, 41, 53, 64].

The task includes 10 neutral faces and 160 faces that express one of four basic emotions (i.e., happiness, sadness, anger, and fear), with pictures morphed between 0% (neutral) and 100% in 10% steps. Two female and two male pictures are used for each of the four emotions. Stimuli are shown in random order for 500 ms and are then replaced by the rating screen where participants have to indicate the correct emotion. The outcome measure is accuracy (proportion correct). The test data is recorded on a computer and processed into scores for each emotion using an established matlab routine according to SOPs (clinical pharmacology, Orca DKF). Accuracy scores are then compared between treatments using ANOVAs for each score. The test is performed once during each of the two main visits and 2-2.5 h after MDMA/placebo administration.

Multifaceted Empathy Test (MET)

The MET is a reliable and valid task to assess the cognitive and emotional aspects of empathy [41, 53, 65]. The MET has been shown to be sensitive to oxytocin and MDMA [67]. The computer-assisted test consists of 40 photographs that showed people in emotionally charged situations. To assess cognitive empathy, the participants are required to infer the mental state of the subject in each scene and indicate the correct mental state from a list of four responses.

Cognitive empathy is defined as the percentage of correct responses in the total responses. To measure emotional empathy, the subjects are asked to rate how much they are feeling for an individual in each scene (i.e., explicit emotional empathy) and how much they are aroused by each scene (i.e., implicit emotional empathy) on a 1-9 point scale. The latter rating provides an inherent additional assessment of emotional empathy, which is considered to reduce the likelihood of socially desirable answers. The three aspects of empathy are each tested with 20 stimuli with positive valence and 20 stimuli with negative valence, resulting in a total of 120 trials. The test requires about 15 minutes. The test data is recorded on a computer and processed into scores for each empathy subscale using an established matlab routine according to SOPs (clinical pharmacology, Orca DKF). Empathy ratings are then compared between treatments using ANOVAs for each score. The test is performed once during each of the two main visits and 2-2.5 h after MDMA/placebo administration.

4.3.3. Telephone interview

Based on previous studies experience no formal follow-up support is needed but we will perform a telephone interview. 3 days after the main visits, patient or healthy participant will receive a phone call to inquire for Adverse Events using:

A. The List of complaints e.g. sleeping problems, dry mouth, dizziness, palpitation, tremor, nausea, headache, transient anxiety and concentrating difficulties.
B. Adverse Events that are not recorded by the LC e.g. subjective signs of depressed mood, psychosomatic problems, psychological problems and any perceptual disturbance e.g. flash backs.

The schedule of events for a subject is shown on **table1**

		**SCREENING VISIT**	**VISIT 1** MDMA/PLACEBO	**VISIT 2** MDMA/PLACEBO	**TELEPHONE INTERVIEW 1** and **2**
Informed consent & Eligibility criteria		x			
**Questionnaires / Examination**					
Medical history questionnaire		x			
General physical examination		x			
ECG / standardized blood pressure		x			
Psychiatric interview (DSM IV)		x			
Questionnaires	Short Form 36 Health Survey (SF 36)	x			
Questionnaires	State Trait Anxiety Inventory (STAI G/S)	x (G)	x(S)	x(S)	
Questionnaires	Toronto Alexythymia Scale (TAS 20)	x			
Questionnaires	Becks Depression Inventory (BDI II)	x			
Questionnaires	List of complaints (LC)		x	x	x
Questionnaires	Visual analog scales (VAS)		x	x	
Face emotion recognition (FERT)			x	x	
Multifaceted empathy task (MET)			x	x	
**Clinical parameters**					
Blood pressure (mmHg)		x	x	x	
Heart rate (bpm)		x	x	x	
Body temperture (°C)		x	x	x	
Weight (kg)		x			
Height (cm)		x			
**Laboratory parameters**					
Red blood cell count		x			
Haemoglobin (g/l)		x			
Plasma sodium (mmol/l)		x	x	x	
Plasma potassium (mmol/l)		x			
Plasma osmolality (mmol/kg)		x			
ALAT (U/l)		x			
ASAT (U/l)		x			
GGT (U/l)		x			
Creatinine (umol/l)		x			
eGFR (ml/min/1,73m2)		x			
MDMA (pg/ml)			x	x	
Oxytocin (ng/ml)			x	x	
Copeptin (pmol/l)			x	x	
Cortisol (nmol/l)			x	x	
ACTH (pg/ml)			x	x	
Prolactin (mIU/l)			x	x	
TSH (mIU/l)			x		
fT4 (pmol/l)			x		
IGF1 (nmol/l)			x		

FSH (IU/l)		x		
LH (IU/l)		x		
Testosterone ♂ (nmol/l) / Oestradiol ♀ (pmol/l)		x		
Urine tests				
Pregnancy test (x)		(x)	(x)	
Drug screening		x	x	

The schedule of session for a subject is shown on **table 2**

	- 60 min	0 min	30 min	60 min	90 min	120 min	150 min	180 min	210 min	240 min	270 min	300 min	330 min	360 min
Short medical questionnaire	x													
**Autonomic measurements**														
Blood pressure (mmHg)	x	x	x	x	x	x	x	x	x	x	x	x	x	x
Heart rate (bpm)	x	x	x	x	x	x	x	x	x	x	x	x	x	x
Body temperature (°C)	x	x	x	x	x	x	x	x	x	x	x	x	x	x
**Laboratory parameters**														
Oxytocin (ng/ml)		x			x	x	x	x				x		
MDMA (pg/ml)		x			x	x	x	x				x		
Copeptin (pmol/l)		x			x		x					x		
Serum cortisol (mmol/l)		x			x		x					x		
Prolactin (umol/l)		x			x		x					x		
ACTH (mmol/l)		x			x		x					x		
Plasma sodium (mmol/l)		x			x	x	x	x				x		
**Screening**														
Drug	x													
Pregnancy	x													
**Psychopathological ratings**														
Face emotion recognition (FERT)							x							
Multifaceted empathy task (MET)							x							
**Questionnaires**														
VAS		x	x	x	x	x	x	x		x		x		x
STAI - S		x						x						
LC		x												x
**Food**														
Standardized Lunch/Breakfast	x							x						

4.4. Withdrawal and discontinuation

If participants do not tolerate the trial procedure the investigator decides whether the participant should be excluded. Participants can at any time withdraw their informed consent to the study. Patients can be excluded from the study due to violation of inclusion criteria or meeting exclusion criteria which were not available at study entry. Withdrawal will be documented in the database with all other study data. If participants withdraw their informed consent to the study, their medical data as well as biological material (blood samples) collected until then, will be analysed and kept in a data blood bank (Biobank Department Endocrinology) in an encrypted/coded fashion. In case of an early study discontinuation, participants will be replaced according to the sample size calculation.

5 STATISTICS AND METHODOLOGY

5.1. Statistical analysis plan and sample size calculation

The null hypothesis is that the area under the concentration time curve of OT is equal for patients with central diabetes insipidus and healthy volunteers after a single dose of MDMA, i.e. the primary endpoint is zero. The alternative hypothesis is that the area under the concentration time of OT is lower in patients with central diabetes insipidus as compared to healthy volunteers, i.e. the primary endpoint is not zero.

The time course of OT measures after intake of MDMA and placebo will be visualized by means of line plots and boxplots for patients with central diabetes insipidus and healthy volunteers. Summary statistics (median, interquartile range, minimum and maximum value) will be presented for each measurement time of OT for MDMA and placebo for patients with central diabetes insipidus and healthy volunteers. Maximum OT time will be summarized for MDMA and placebo for patients with central diabetes insipidus and healthy volunteers separately by means of frequency distribution according to the measurement times, and by median, interquartile range, minimum and maximum value. OT area under the concentration time curve from baseline to 6 hours after intervention will be tested for a difference between MDMA and placebo for patients with central diabetes insipidus and healthy volunteers. Summary statistics (median, interquartile range, minimum and maximum value) will be presented separately.

All other secondary endpoints will be analyzed descriptively. The time course of MDMA, cortisol, prolactin, copeptin, and ACTH levels will be visualized by means of line plots and boxplots, and summary statistics will be presented for each measurement time for MDMA and placebo for patients with central diabetes insipidus and healthy volunteers separately.

Sample size was estimated in order to show a significant increase in OT after the intake of a single oral dose of MDMA with a power of 90 % and a significance level α of 0.05. Sample size estimation was based on preliminary data from 28 healthy adults. In this trial, OT levels following MDMA intake was studied. The mean increase in OT levels for MDMA was 809+/-64 pg/mL and 259 +/- 62 pmol for placebo.

Assuming 30% reduced levels of OT after MDMA in patients with central diabetes insipidus as compared to healthy volunteers, a total of 15 subjects should be recruited in order to have N = 12 evaluable subjects, assuming a drop-out rate of 20 %.

5.2. Handling of missing data and drop-outs

No data imputation is foreseen for missing data. Subjects will be enrolled and any drop-outs during the study replaced to reach a final study sample of at least 30 subjects. Any drop-out will be replaced by recruitment of new subjects.

6 REGULATORY ASPECTS AND SAFETY

6.1. Local regulations / Declaration of Helsinki

This study is conducted in compliance with the protocol, the current version of the Declaration of Helsinki, the ICH-GCP the HRA as well as other locally relevant legal and regulatory requirements.

6.2. (Serious) Adverse Events

An **Adverse Event (AE)** is any untoward medical occurrence in a patient or a clinical investigation subject which does not necessarily have a causal relationship with the trial procedure. An AE can therefore be any unfavourable or unintended finding, symptom, or disease temporally associated with a trial procedure, whether or not related to it.

A Serious Adverse Event (SAE) (ClinO, Art. 63) is any untoward medical occurrence that

- Results in death or is life-threatening,
- Requires in-patient hospitalization or prolongation of existing hospitalization,
- Results in persistent or significant disability or incapacity, or
- Causes a congenital anomaly or birth defect

Both Investigator and Sponsor-Investigator make a causality assessment of the event to the trial invervention, (see table below based on the terms given in ICH E2A guidelines). Any event assessed as possibly, probably or definitely related is classified as related to the trial intervention.

Relationship	Description
Definitely	Temporal relationship<br>Improvement after dechallenge*<br>Recurrence after rechallenge<br>(or other proof of drug cause)
Probably	Temporal relationship<br>Improvement after dechallenge<br>No other cause evident
Possibly	Temporal relationship<br>Other cause possible
Unlikely	Any assessable reaction that does not fulfil the above conditions
Not related	Causal relationship can be ruled out
*Improvement after dechallenge only taken into consideration, if applicable to reaction	

Both Investigator and Sponsor-Investigator make a severity assessment of the event as mild, moderate or severe. Mild means the complication is tolerable, moderate means it interferes with daily activities and severe means it renders daily activities impossible.

Reporting of SAEs (see ClinO, Art. 63)
All SAEs are documented and reported immediately (within a maximum of 24 hours) to the Sponsor-Investigator of the study.
If it cannot be excluded that the SAE occurring in Switzerland is attributable to the intervention under investigation, the Investigator reports it to the Ethics Committee via BASEC within 15 days.

Follow up of (Serious) Adverse Events
Following a comprehensive baseline evaluation, each patient's safety will be monitored with periodic record and evaluation of all treatment-emergent AEs.
One evidence of a clinical abnormality is noticed, the condition will be treated while trying to determine its cause. The patient will then be followed until the condition resoles or becomes chronic or stable. Patients will be instructed about possible acute AEs. If adverse events are observed, the participants are promptly treated according to standard of care.

6.3. (Periodic) safety reporting

An annual safety report (ASR/DSUR) is submitted once a year to the local Ethics Committee by the Investigator (ClinO, Art. 43 Abs).

6.4. Radiation

N/A.

6.5. Pregnancy

In case of pregnancy, study visit will not be started (see exclusion criteria). Pregnancies during the study will be reported within a maximum of 24 hours to the Sponsor-Investigator, and patient is withdrawn, and outcome of the pregnancy is followed up. An impairment of the sperm cells cannot be ruled out; therefore, patients and healthy participants must use a double contraceptive method (e.g. condom and pill) during the study and until one month afterwards.

6.6. Amendments

Substantial changes to the study setup and study organization, the protocol and relevant study documents are submitted to the Ethics Committee for approval before implementation. Under emergency circumstances, deviations from the protocol to protect the rights, safety and well-being of human subjects may proceed without prior approval of the Ethics Committee. Such deviations shall be documented and reported to the Ethics Committee as soon as possible.

Substantial amendments are changes that affect the safety, health, rights and obligations of participants, changes in the protocol that affect study objective(s) or central research topic, changes of study site(s) or of study leader and sponsor (ClinO, Art. 29).

6.7. (Premature) termination of study

The Sponsor-Investigator may terminate the study prematurely according to certain circumstances, e.g.

- Ethical concerns,
- Insufficient participant recruitment,
- When the safety of the participants is doubtful or at risk (e.g. when the benefit-risk assessment is no longer positive),
- Alterations in accepted clinical practice that make the continuation of the study unwise, or
- Early evidence of harm or benefit of the experimental intervention

Upon regular study termination, the Ethics Committee is notified via BASEC within 90 days (ClinO, Art. 38).
Upon premature study termination or study interruption, the Ethics Committee is notified via BASEC within 15 days (ClinO, Art. 38).
Please refer to www.swissethics.ch for a template concerning the notification of completion, discontinuation or interruption of the clinical trial.

All biological materials and health-related data will not be anonymized upon end of data analysis.

6.8. Insurance

Insurance will be provided by the Sponsor. A copy of the insurance certificate is filled in the investigator site file and trial master file.

7 FURTHER ASPECTS

7.1. Overall ethical considerations

This study will identify the role of OT in patients with central diabetes insipidus. Recent findings have suggested low OT levels in relation to increased psychopathology in patients with central diabetes insipidus. However, OT measurements are difficult, and no provocation test is yet available. This study will improve the pathophysiological implications of OT in patients with central diabetes insipidus by clarifying whether these patients indeed have OT deficiency. As standard OT measurements are unreliable and as for other pituitary hormones provocation tests are used, this study will explore whether MDMA can be used as provocation test for OT in patients with central diabetes insipidus and healthy controls. Future studies could consider interventional studies using OT application to reduce pathophysiology in patients with central diabetes insipidus and ultimately increase their quality of life.

The cross-over design with a washout period of at least two weeks is considered appropriate as first, no residual effects of MDMA is expected to be present after two weeks. Furthermore, the cross-over design allows a lower sample size as for the reduced inter-individual variability. In order to increase the understanding of the pathophysiology of OT and MDMA in patients with central diabetes insipidus it is crucial to compare the values to healthy controls. Only with these two study populations can we conclude how OT is stimulated following MDMA and whether an OT deficiency is present in patients with central diabetes insipidus.

7.2. Risk-benefit assessment

Trained study personnel will place venous accesses, perform intervention and collect blood samples. The frequency of the following possible side effects corresponds to the international guidelines of MedDRA. “Very common” corresponds to (≥10%), “common” (1-10%), “uncommon” (0.1-1%), “rare” 1/1000-1/10’000), and “very rare” (<1/10’000).

Blood sampling: At the injection sites for the venous catheter, small bruises can occur, but these will heal in a few days. In rare cases, vein occlusion or inflammation of the injection site may occur. If this is the case, the patient or healthy participant will be asked to report this immediately.

Psychopathological questionnaires: Patients or healthy participants do not have to answer any of the questions asked if they do not want to. If there is an emotional burden during the session /in-between the sessions or if there is a need for information or advice, the study physician can be contacted for further information and support.

MDMA: Several studies in healthy subjects with a single dose of MDMA has been conducted frequently and shown to be safe in controlled settings [33, 34, 38, 43, 51, 68–71]. This

considerable body of evidence indicates that the likelihood for significant toxicity from doses of MDMA such as the one to be used in the present study and in controlled settings is very low. Common moderate acute adverse effects can be increased jaw muscle tension, dry mouth, dizziness, palpitations, tremor, nausea, headache, insomnia and transient anxiety in approximately 10% of subjects [43, 69, 72].

The main expected acute adverse effects are consistent with moderate sympathomimetic toxicity including moderate hypertension, tachycardia and a raise in body temperature. Patients and healthy participants will be screened with an ECG and standardized blood pressure measurement; any pathological finding will be assessed by the study physician. Closer monitoring will be implemented if blood pressure values exceed 180/120 mmHg systolic blood pressure (P_{sys}). Treatment of a hypertensive reaction (P_{sys}>220 mmHg) may include administration of oxygen, nitrates and nifedipine, or esmolol and/or transfer to medical services (Emergency Department). In the case of angina pectoris, oxygen is given, and ECG monitoring is initiated, and cardiac enzymes will be measured followed by consultation with the cardiologist on call. Cardiac arrest triggers immediate cardiopulmonary reanimation and defibrillation by the study team (defibrillator on site) and call to the ambulance (0144). An increase in body temperature of 0.5°C are expected after MDMA administration [43, 72, 73]. If body temperature rises by more than 1.5°C appropriate treatment including hydration and cooling will be initiated [56].

Ecstasy use has been associated with both dehydration and hyperhydration leading to hyponatremia [56, 74–77]. Subjects will be encouraged to drink to thirst, but in general no less than 1 and no more than 3 l during one session. Serum sodium levels checked as described in Table 2 at timepoint 0min, 90min, 120min, 150min, 180min and 300min. If symptoms of hyponatremia such as confusion or vomiting present between these intervals, subjects will be further examined and sodium levels rechecked. Participants will be informed of these symptoms and encouraged to report if these symptoms occur following the study visit.
Acute psychological distress was observed to be minimal; no lasting biological or psychological injury is expected. There is no indication that administration of MDMA in a controlled setting has any adverse effects on cognitive function [30, 72, 85].

Recreational use of Ecstasy has been linked to reversible neurotoxicity [78, 79], especially when the drug is taken frequently and in high doses [80]. In contrast, imaging studies do not indicate any changes after moderate MDMA use [35]. Several studies document no lasting effects of Ecstasy in moderate Ecstasy users [53, 78, 81]. No changes in serotonin transporter density were seen using positron emission tomography and [11C]-McN5652 four weeks after MDMA

administration (1.5-1.7 mg/kg) in MDMA-naive human volunteers [86,87]. The use of MDMA in controlled settings is considered safe with regard to neurotoxic effects [47, 82].

MDMA has rarely been associated with liver toxicity but often under overdose and not in controlled clinical setting [56]. Liver enzymes will be measured at the screening visit and pathological results will be excluded regarding the exclusion criteria.

The participants will be under continuous medical supervision and closely monitored during MDMA application and until the acute effects completely subsided; expected within 6h for MDMA [74]. Subjects with known risk factors for future drug abuse including current major depression, psychotic disorder and anxiety disorders [83] and prior drug dependence or other relevant illicit drug use [84] will be excluded from the study. Illicit drug use will also be monitored during the study using repeated urine drug screens before both main sessions.

Six hours after stimulation participants will be allowed to return to their home alone. If effects persist, the night is spent at the research facility. In the case of unexpected prolonged or severe adverse reactions subjects will remain in the hospital and are monitored as long as necessary. Subjects will be provided with the phone number of the study physicians in case of an emergency between/after study sessions.
MDMA is expected to produce sequelae (1-3 days post MDMA) including difficulty concentrating, irritability, or slightly depressed mood in about 10% of subjects [69]. Participants will receive a phone call 3 days after the intervention to asses mood changes and adverse events.

MDMA is neither mutagenic nor teratogenic and their chronic use is not associated with birth defects. Pregnant women are excluded from the study and pregnancy tests will be done before each test session. Effective birth control will be mandatory for female participants.

We do not expect immediate benefit to the study participants. Our data could clarify an OT deficiency in patients with central diabetes insipidus and give new information for further studies.

8 QUALITY CONTROL AND DATA PROTECTION

8.1. Quality measures

For quality assurance the sponsor, the Ethics Committee or an independent trial monitor may visit the research sites. Direct access to the source data and all study related files is granted on such occasions. All involved parties keep the participant data strictly confidential.

8.2. Data recording and source data

electronic Case Report Forms

Study data will be recorded in Source Documents and transferred to electronic Case Report Forms (eCRF) in an encrypted fashion by their individual study participant number provided by CTU Basel. For each enrolled study participant, the eCRF must be kept current to reflect subject status at each phase during the course of study. Site Principal Investigators and, if applicable, delegates at each site will be authorized to do eCRF entries.

Specification of source documents

Source data will be available to document the existence of the study participants. Source data will include the original documents relating to the study:

The written informed consent forms, demographic data of the patients, the study number, the visit dates and results of laboratory analysis. The individual source data will be stored inside an Investigator Site File (ISF).

The investigator will permit study related monitoring visits, audits, ethics committee reviews, and regulatory inspections, and provide direct access to all source data.

Screening information containing personal information of the volunteers is kept separately to ensure the blinding from screening to enrolment. Further, screening numbers will not reflect the latter subject number in the study. Additionally, enrolment logs will be anonymized after the monitoring.

Record keeping / archiving

Records and documents pertaining the conduct of this study, including eCRFs, consent forms, laboratory test results, clinical notes and study datasets will be retained for 10 years.

8.3. Confidentiality and coding

Direct access to source documents will be permitted for purposes of monitoring, audits and inspections. The investigators of the study will have access to the protocol and the dataset. The statistician will have access to the statistical code during and after the study. The participant identities will never be published in any abstracts or publications. A transfer of data will only take place for study purposes and only in encoded form. Third persons will not gain any insight into

source data. For inspection purposes, insight to source data will be permitted to the member of the appropriate authorities and also for members of the local ethics committee. During the study, confidentiality will be guaranteed. The principal investigator will guarantee for compliance with national and international data security.
Study data entered into the eCRF are only accessible by authorized persons. Once all data are entered into the EDC system and monitoring is completed, the database will be locked and closed for further data entry. The complete dataset is then exported and transferred to the study statistician as well as the principal investigator through a secure channel. On the eCRFs and other study specific documents, participants are only identified by a unique participant number.

8.4. Retention and destruction of study data and biological material

Biological material, i.e. blood samples, will be handled according to good clinical practice and good laboratory practice. Samples will be collected in secure containers (Sarstedt Monovette®) and will be centrifuged to collect serum. Serum will be stored at – 80°C in a thermo-controlled ultra-deep freezer. Data and samples are stored for eventual future research aims (Biobank Department Endocrinology). Records and documents pertaining the conduct of this study, including eCRFs, consent forms, laboratory test results and clinical notes will be retained for 10 years.

9 MONITORING AND REGISTRATION

9.1. Monitoring

The study will be monitored by the Clinical Trial Unit Basel. Regular monitoring will take place at the investigator's site during the course of the study, as organized by the Sponsor. The extent and nature of monitoring activities based on the objective and design of the study are defined in a study specific monitoring plan. To this purpose, source data/documents are made accessible to monitors and questions are answered during monitoring.

9.2. Registration

The study will be registered at www.clinicaltrials.gov and the Swiss National Clinical Trial Portal www.kofam.ch.

10 FUNDING / PUBLICATION / DECLARATION OF INTEREST

10.1. Budget and Funding

Costs are covered by the University Hospital, the Department of Endocrinology, Diabetes and Metabolism. The researchers do not receive any payments for the conduct of this research besides from their salaries.

10.2. Publications

All results will be presented at congress meetings and published in peer-reviewed scientific journals.

10.3. DECLARATION OF INTEREST

The Sponsor-Investigator has no conflicts of interest to declare.

10 REFERENCES

[1] I. Crespo, A. Santos, and S. M. Webb, "Quality of life in patients with hypopituitarism," *Curr. Opin. Endocrinol. Diabetes Obes.*, vol. 22, no. 4, pp. 306–312, 2015, doi:10.1097/MED.0000000000000169.

[2] D. Mo, W. F. Blum, M. Rosilio, S. M. Webb, R. Qi, and C. J. Strasburger, "Ten-year change in quality of life in adults on growth hormone replacement for growth hormone deficiency: An analysis of the hypopituitary control and complications study," *J. Clin. Endocrinol. Metab.*, vol. 99, no. 12, pp. 4581–4588, 2014, doi: 10.1210/jc.2014-2892.

[3] O. M. Dekkers *et al.*, "Quality of life in treated adult craniopharyngioma patients," *Eur. J. Endocrinol.*, vol. 154, no. 3, pp. 483–489, 2006, doi: 10.1530/eje.1.02114.

[4] E. H. Nielsen *et al.*, "Nonfunctioning pituitary adenoma: Incidence, causes of death and quality of life in relation to pituitary function," *Pituitary*, vol. 10, no. 1, pp. 67–73, 2007, doi: 10.1007/s11102-007-0018-x.

[5] R. K. Crowley *et al.*, "Morbidity and mortality in patients with craniopharyngioma after surgery," *Clin. Endocrinol. (Oxf).*, vol. 73, no. 4, pp. 516–521, 2010, doi: 10.1111/j.1365-2265.2010.03838.x.

[6] C. Baskaran *et al.*, "Oxytocin secretion is pulsatile in men and is related to social-emotional functioning," *Psychoneuroendocrinology*, vol. 85, no. January, pp. 28–34, 2017, doi: 10.1016/j.psyneuen.2017.07.486.

[7] D. A. Mechanism, F. Determine, T. H. E. Intermittent, and D. Suckling, "Of oxytocin," 1973.

[8] H. Walum *et al.*, "Variation in the oxytocin receptor gene is associated with pair-bonding and social behavior," *Biol. Psychiatry*, vol. 71, no. 5, pp. 419–426, 2012, doi: 10.1016/j.biopsych.2011.09.002.

[9] C. S. Carter *et al.*, "Oxytocin: Behavioral associations and potential as a salivary biomarker," *Ann. N. Y. Acad. Sci.*, vol. 1098, pp. 312–322, 2007, doi: 10.1196/annals.1384.006.

[10] S. E. E. L. Page, "Oxytocin.Vasopressin.Neurogenetics," vol. 322, no. November 2008, pp. 900–905, 2009.

[11] R. Feldman, I. Gordon, and O. Zagoory-Sharon, "The cross-generation transmission of oxytocin in humans," *Horm. Behav.*, vol. 58, no. 4, pp. 669–676, 2010, doi: 10.1016/j.yhbeh.2010.06.005.

[12] R. Feldman and M. J. Bakermans-Kranenburg, "Oxytocin: a parenting hormone," *Curr. Opin. Psychol.*, vol. 15, pp. 13–18, 2017, doi: 10.1016/j.copsyc.2017.02.011.

[13] T. R. Insel, B. S. Gingrich, and L. J. Young, "Oxytocin: Who needs it?," *Prog. Brain Res.*, vol. 133, pp. 59–66, 2001, doi: 10.1016/S0079-6123(01)33005-4.

[14] T. R. Insel and L. J. Young, "The neurobiology of attachment," *Nat. Rev. Neurosci.*, vol. 2, no. 2, pp. 129–136, 2001, doi: 10.1038/35053579.

[15] K. MacDonald and T. M. MacDonald, "The peptide that binds: A systematic review of Oxytocin and its prosocial effects in humans," *Harv. Rev. Psychiatry*, vol. 18, no. 1, pp. 1–21, 2010, doi: 10.3109/10673220903523615.

[16] W. Mark, "Very long-term sequelae of craniopharyngioma," vol. 31, no. March, pp. 1–26, 2017.

[17] A. Aulinas *et al.*, "Low Plasma Oxytocin Levels and Increased Psychopathology in Hypopituitary Men with Diabetes Insipidus," *J. Clin. Endocrinol. Metab.*, vol. 104, no. 8, pp. 3181–3191, 2019, doi: 10.1210/jc.2018-02608.

[18] K. Daughters, A. S. R. Manstead, and D. A. Rees, "Hypopituitarism is associated with lower oxytocin concentrations and reduced empathic ability," *Endocrine*, vol. 57, no. 1, pp. 166–174, 2017, doi: 10.1007/s12020-017-1332-3.

[19] Y. Eisenberg *et al.*, "Oxytocin alterations and neurocognitive domains in patients with hypopituitarism," *Pituitary*, vol. 22, no. 2, pp. 105–112, 2019, doi: 10.1007/s11102-019-00936-0.

[20] G. Leng and N. Sabatier, "Measuring Oxytocin and Vasopressin: Bioassays, Immunoassays and Random Numbers," *J. Neuroendocrinol.*, vol. 28, no. 10, 2016, doi: 10.1111/jne.12413.

[21] M. E. McCullough, P. S. Churchland, and A. J. Mendez, "Problems with measuring peripheral oxytocin: Can the data on oxytocin and human behavior be trusted?," *Neurosci. Biobehav. Rev.*, vol. 37, no. 8, pp. 1485–1492, 2013, doi: 10.1016/j.neubiorev.2013.04.018.
[22] A. M. M. Daubenbüchel *et al.*, "Oxytocin in survivors of childhood-onset craniopharyngioma," *Endocrine*, vol. 54, no. 2, pp. 524–531, 2016, doi: 10.1007/s12020-016-1084-5.
[23] J. Martin, S. M. Kagerbauer, J. Gempt, A. Podtschaske, A. Hapfelmeier, and G. Schneider, "Oxytocin levels in saliva correlate better than plasma levels with concentrations in the cerebrospinal fluid of patients in neurocritical care," *J. Neuroendocrinol.*, vol. 30, no. 5, pp. 0–1, 2018, doi: 10.1111/jne.12596.
[24] D. S. Carson *et al.*, "Cerebrospinal fluid and plasma oxytocin concentrations are positively correlated and negatively predict anxiety in children," *Mol. Psychiatry*, vol. 20, no. 9, pp. 1085–1090, 2015, doi: 10.1038/mp.2014.132.
[25] S. M. Francis, M. G. Kirkpatrick, H. de Wit, and S. Jacob, "Urinary and plasma oxytocin changes in response to MDMA or intranasal oxytocin administration," *Psychoneuroendocrinology*, vol. 74, pp. 92–100, 2016, doi: 10.1016/j.psyneuen.2016.08.011.
[26] A. Szeto *et al.*, "Evaluation of enzyme immunoassay and radioimmunoassay methods for the measurement of plasma oxytocin," *Psychosom. Med.*, vol. 73, no. 5, pp. 393–400, 2011, doi: 10.1097/PSY.0b013e31821df0c2.
[27] P. Chiodera *et al.*, "Oxytocin response to challenging stimuli in elderly men," *Regul. Pept.*, vol. 51, no. 2, pp. 169–176, 1994, doi: 10.1016/0167-0115(94)90206-2.
[28] P. Chiodera, R. Volpi, L. Capretti, N. Giuliani, G. Caffarri, and V. Coiro, "Melatonin inhibits oxytocin response to insulin-induced hypoglycemia, but not to angiotensin II in normal men," *J. Neural Transm.*, vol. 105, no. 2–3, pp. 173–180, 1998, doi: 10.1007/s007020050046.
[29] A. A. Feduccia and M. C. Mithoefer, "MDMA-assisted psychotherapy for PTSD: Are memory reconsolidation and fear extinction underlying mechanisms?," *Prog. Neuro-Psychopharmacology Biol. Psychiatry*, vol. 84, no. February, pp. 221–228, 2018, doi: 10.1016/j.pnpbp.2018.03.003.
[30] B. Sessa and D. J. Nutt, "MDMA, politics and medical research: Have we thrown the baby out with the bathwater?," *J. Psychopharmacol.*, vol. 21, no. 8, pp. 787–791, 2007, doi: 10.1177/0269881107084738.
[31] B. Sessa, "MDMA and PTSD treatment: 'PTSD: From novel pathophysiology to innovative therapeutics,'" *Neurosci. Lett.*, vol. 649, pp. 176–180, 2017, doi: 10.1016/j.neulet.2016.07.004.
[32] M. C. Mithoefer *et al.*, "MDMA-assisted psychotherapy for treatment of PTSD: study design and rationale for phase 3 trials based on pooled analysis of six phase 2 randomized controlled trials," *Psychopharmacology (Berl).*, vol. 236, no. 9, pp. 2735–2745, 2019, doi: 10.1007/s00213-019-05249-5.
[33] F. X. Vollenweider, A. Gamma, M. Liechti, and T. Huber, "Psychological and cardiovascular effects and short-term sequelae of MDMA ('Ecstasy') in MDMA-naive healthy volunteers," *Neuropsychopharmacology*, vol. 19, no. 4, pp. 241–251, 1998, doi: 10.1016/S0893-133X(98)00013-X.
[34] F. X. Vollenweider, M. E. Liechti, A. Gamma, G. Greer, and M. Geyer, "Acute psychological and neurophysiological effects of MDMA in humans," *J. Psychoactive Drugs*, vol. 34, no. 2, pp. 171–184, 2002, doi: 10.1080/02791072.2002.10399951.
[35] F. Mueller *et al.*, "Neuroimaging in moderate MDMA use: A systematic review," *Neurosci. Biobehav. Rev.*, vol. 62, pp. 21–34, 2016, doi: 10.1016/j.neubiorev.2015.12.010.
[36] R. L. Carhart-Harris *et al.*, "The effects of acutely administered 3,4-methylenedioxymethamphetamine on spontaneous brain function in healthy volunteers measured with arterial spin labeling and blood oxygen level-dependent resting state functional connectivity," *Biol. Psychiatry*, vol. 78, no. 8, pp. 554–562, 2015, doi: 10.1016/j.biopsych.2013.12.015.
[37] G. J. H. Dumont *et al.*, "Increased oxytocin concentrations and prosocial feelings in humans after ecstasy (3,4-methylenedioxymethamphetamine) administration," *Soc. Neurosci.*, vol. 4, no. 4, pp. 359–366, 2009, doi: 10.1080/17470910802649470.
[38] C. M. Hysek and M. E. Liechti, "Effects of MDMA alone and after pretreatment with reboxetine, duloxetine, clonidine, carvedilol, and doxazosin on pupillary light reflex," *Psychopharmacology (Berl).*, vol. 224, no. 3, pp. 363–376, 2012, doi: 10.1007/s00213-012-2761-6.
[39] C. M. Hysek *et al.*, "Pharmacokinetic and pharmacodynamic effects of methylphenidate and MDMA administered alone or in combination," *Int. J. Neuropsychopharmacol.*, vol. 17, no. 3, pp. 371–381, 2014, doi: 10.1017/S1461145713001132.
[40] M. G. Kirkpatrick, R. Lee, M. C. Wardle, S. Jacob, and H. De Wit, "Effects of MDMA and intranasal oxytocin on social and emotional processing," *Neuropsychopharmacology*, vol. 39, no. 7, pp. 1654–1663, 2014, doi: 10.1038/npp.2014.12.

[41] Y. Schmid, C. M. Hysek, L. D. Simmler, M. J. Crockett, B. B. Quednow, and M. E. Liechti, “Differential effects of MDMA and methylphenidate on social cognition,” *J. Psychopharmacol.*, vol. 28, no. 9, pp. 847–856, 2014, doi: 10.1177/0269881114542454.
[42] P. Vizeli and M. E. Liechti, “Oxytocin receptor gene variations and socio-emotional effects of MDMA: A pooled analysis of controlled studies in healthy subjects,” *PLoS One*, vol. 13, no. 6, pp. 10–14, 2018, doi: 10.1371/journal.pone.0199384.
[43] P. Vizeli and M. E. Liechti, “Safety pharmacology of acute MDMA administration in healthy subjects,” *J. Psychopharmacol.*, vol. 31, no. 5, pp. 576–588, 2017, doi: 10.1177/0269881117691569.
[44] C. M. Hysek *et al.*, “The norepinephrine transporter inhibitor reboxetine reduces stimulant effects of MDMA (ecstasy) in humans,” *Clin. Pharmacol. Ther.*, vol. 90, no. 2, pp. 246–255, 2011, doi: 10.1038/clpt.2011.78.
[45] P. Oehen, R. Traber, V. Widmer, and U. Schnyder, “A randomized, controlled pilot study of MDMA (±3,4- Methylenedioxymethamphetamine)-assisted psychotherapy for treatment of resistant, chronic Post-Traumatic Stress Disorder (PTSD),” *J. Psychopharmacol.*, vol. 27, no. 1, pp. 40–52, 2013, doi: 10.1177/0269881112464827.
[46] M. C. Mithoefer, M. T. Wagner, A. T. Mithoefer, L. Jerome, and R. Doblin, “The safety and efficacy of ±3,4-methylenedioxymethamphetamine- assisted psychotherapy in subjects with chronic, treatment-resistant posttraumatic stress disorder: The first randomized controlled pilot study,” *J. Psychopharmacol.*, vol. 25, no. 4, pp. 439–452, 2011, doi: 10.1177/0269881110378371.
[47] F. X. Vollenweider, A. Gamma, M. Liechti, and T. Huber, “Is a single dose of MDMA harmless? [2],” *Neuropsychopharmacology*, vol. 21, no. 4, pp. 598–600, 1999, doi: 10.1016/S0893-133X(99)00032-9.
[48] E. Kolbrich and R. Goodwin, “Physiological and subjective responses to controlled oral MDMA administration,” *J. Clin. …*, vol. 28, no. 4, pp. 432–440, 2008, doi: 10.1097/JCP.0b013e31817ef470.Physiological.
[49] E. A. Kolbrich, R. S. Goodwin, D. A. Gorelick, R. J. Hayes, E. A. Stein, and M. A. Huestis, “Plasma Pharmacokinetics of 3,4-Methylenedioxymethamphetamine after Controlled Oral Administration to Young Adults,” *Ther. Drug Monit.*, vol. 30, no. 3, pp. 320–332, 2008, doi: 10.1097/FTD.0b013e3181684fa0.
[50] R. De La Torre *et al.*, “Non-linear pharmacokinetics of MDMA ('ecstasy’) in humans,” *Br. J. Clin. Pharmacol.*, vol. 49, no. 2, pp. 104–109, 2000, doi: 10.1046/j.1365-2125.2000.00121.x.
[51] R. De La Torre *et al.*, “Human Pharmacology of MDMA: Pharmacokinetics, Metabolism, and Disposition,” *Ther. Drug Monit.*, vol. 26, no. 2, pp. 137–144, 2004, doi: 10.1097/00007691-200404000-00009.
[52] R. Bhargava, K. L. Daughters, and A. Rees, “Oxytocin therapy in hypopituitarism: Challenges and opportunities,” *Clin. Endocrinol. (Oxf).*, vol. 90, no. 2, pp. 257–264, 2019, doi: 10.1111/cen.13909.
[53] J. H. Halpern, H. G. Pope, A. R. Sherwood, S. Barry, J. I. Hudson, and D. Yurgelun-Todd, “Residual neuropsychological effects of illicit 3,4- methylenedioxymethamphetamine (MDMA) in individuals with minimal exposure to other drugs,” *Drug Alcohol Depend.*, vol. 75, no. 2, pp. 135–147, 2004, doi: 10.1016/j.drugalcdep.2004.02.008.
[54] C. M. Hysek *et al.*, “MDMA enhances emotional empathy and prosocial behavior,” *Soc. Cogn. Affect. Neurosci.*, vol. 9, no. 11, pp. 1645–1652, 2014, doi: 10.1093/scan/nst161.
[55] Y. Schmid *et al.*, “Effects of methylphenidate and MDMA on appraisal of erotic stimuli and intimate relationships,” *Eur. Neuropsychopharmacol.*, vol. 25, no. 1, pp. 17–25, 2015, doi: 10.1016/j.euroneuro.2014.11.020.
[56] M. E. Liechti, “Behandlung der akuten Intoxikation,” no. 7, 2003.
[57] M. W. Johnson, W. A. Richards, and R. R. Griffiths, “Human hallucinogen research: Guidelines for safety,” *J. Psychopharmacol.*, vol. 22, no. 6, pp. 603–620, 2008, doi: 10.1177/0269881108093587.
[58] L. J. Julian, “Measures of anxiety: State-Trait Anxiety Inventory (STAI), Beck Anxiety Inventory (BAI), and Hospital Anxiety and Depression Scale-Anxiety (HADS-A),” *Arthritis Care Res.*, vol. 63, no. SUPPL. 11, pp. 467–472, 2011, doi: 10.1002/acr.20561.
[59] C. Treanor and M. Donnelly, “A methodological review of the Short Form Health Survey 36 (SF-36) and its derivatives among breast cancer survivors,” *Qual. Life Res.*, vol. 24, no. 2, pp. 339–362, 2015, doi: 10.1007/s11136-014-0785-6.
[60] S. Torres *et al.*, “Factorial Validity of the Toronto Alexithymia Scale (TAS-20) in Clinical Samples: A Critical Examination of the Literature and a Psychometric Study in Anorexia Nervosa,” *J. Clin. Psychol. Med. Settings*, vol. 26, no. 1, pp. 33–46, 2019, doi: 10.1007/s10880-018-9562-y.
[61] R. M. Bagby, J. D. A. Parker, and G. J. Taylor, “Twenty-five years with the 20-item Toronto Alexithymia Scale,” *J. Psychosom. Res.*, vol. 131, no. December 2019, p. 109940, 2020, doi:

10.1016/j.jpsychores.2020.109940.
[62] H. S. Paul Richter, Joachim Werner, Andrés Heerlein, Alfred Kraus, "On the Validity of the Beck Depression," *Psychopathology*, pp. 160–168, 1998.
[63] G. Jackson-Koku, "Beck depression inventory," *Occup. Med. (Chic. Ill).*, vol. 66, no. 2, pp. 174–175, 2016, doi: 10.1093/occmed/kqv087.
[64] C. M. Hysek, G. Domes, and M. E. Liechti, "MDMA enhances 'mind reading' of positive emotions and impairs 'mind reading' of negative emotions," *Psychopharmacology (Berl).*, vol. 222, no. 2, pp. 293–302, 2012, doi: 10.1007/s00213-012-2645-9.
[65] G. Bedi, D. Hyman, and H. De Wit, "Is ecstasy an 'empathogen'? Effects of ±3,4-methylenedioxymethamphetamine on prosocial feelings and identification of emotional states in others," *Biol. Psychiatry*, vol. 68, no. 12, pp. 1134–1140, 2010, doi: 10.1016/j.biopsych.2010.08.003.
[66] I. Dziobek *et al.*, "Dissociation of cognitive and emotional empathy in adults with Asperger syndrome using the Multifaceted Empathy Test (MET)," *J. Autism Dev. Disord.*, vol. 38, no. 3, pp. 464–473, 2008, doi: 10.1007/s10803-007-0486-x.
[67] R. Hurlemann *et al.*, "Oxytocin enhances amygdala-dependent, socially reinforced learning and emotional empathy in humans," *J. Neurosci.*, vol. 30, no. 14, pp. 4999–5007, 2010, doi: 10.1523/JNEUROSCI.5538-09.2010.
[68] A. Gamma, A. Buck, T. Berthold, D. Hell, and F. X. Vollenweider, "3,4-Methylenedioxymethamphetamine (MDMA) modulates cortical and limbic brain activity as measured by [H215O]-PET in healthy humans," *Neuropsychopharmacology*, vol. 23, no. 4, pp. 388–395, 2000, doi: 10.1016/S0893-133X(00)00130-5.
[69] M. E. Liechti and F. X. Vollenweider, "The serotonin uptake inhibitor citalopram reduces acute cardiovascular and vegetative effects of 3,4-methylenedioxymethamphetamine ('Ecstasy') in healthy volunteers," *J. Psychopharmacol.*, vol. 14, no. 3, pp. 269–274, 2000, doi: 10.1177/026988110001400313.
[70] M. Tancer and C. E. Johanson, "Reinforcing, subjective, and physiological effects of MDMA in humans: A comparison with d-amphetamine and mCPP," *Drug Alcohol Depend.*, vol. 72, no. 1, pp. 33–44, 2003, doi: 10.1016/S0376-8716(03)00172-8.
[71] J. Camarasa, J. M. Marimón, T. Rodrigo, E. Escubedo, and D. Pubill, "Memantine prevents the cognitive impairment induced by 3,4-methylenedioxymethamphetamine in rats," *Eur. J. Pharmacol.*, vol. 589, no. 1–3, pp. 132–139, 2008, doi: 10.1016/j.ejphar.2008.05.014.
[72] M. E. Liechti, A. Gamma, and F. X. Vollenweider, "Gender differences in the subjective effects of MDMA," *Psychopharmacology (Berl).*, vol. 154, no. 2, pp. 161–168, 2001, doi: 10.1007/s002130000648.
[73] M. E. Liechti, "Effects of MDMA on body temperature in humans," *Temperature*, vol. 1, no. 3, pp. 192–200, 2014, doi: 10.4161/23328940.2014.955433.
[74] L. D. Simmler, C. M. Hysek, and M. E. Liechti, "Sex differences in the effects of MDMA (ecstasy) on plasma copeptin in healthy subjects," *J. Clin. Endocrinol. Metab.*, vol. 96, no. 9, pp. 2844–2850, 2011, doi: 10.1210/jc.2011-1143.
[75] J. Rosenson, C. Smollin, K. A. Sporer, P. Blanc, and K. R. Olson, "Patterns of Ecstasy-Associated Hyponatremia in California," *Ann. Emerg. Med.*, vol. 49, no. 2, 2007, doi: 10.1016/j.annemergmed.2006.09.018.
[76] A. P. Hall and J. A. Henry, "Acute toxic effects of 'Ecstasy' (MDMA) and related compounds: Overview of pathophysiology and clinical management," *Br. J. Anaesth.*, vol. 96, no. 6, pp. 678–685, 2006, doi: 10.1093/bja/ael078.
[77] D. L. Maxwell, M. I. Polkey, and J. A. Henry, "Hyponatraemia and catatonic stupor after taking 'ecstasy,'" *Br. Med. J.*, vol. 307, no. 6916, p. 1399, 1993, doi: 10.1136/bmj.307.6916.1399.
[78] L. Reneman *et al.*, "Effects of dose, sex, and long-term abstention from use on toxic effects of MDMA (ecstasy) on brain serotonin neurons," *Lancet*, vol. 358, no. 9296, pp. 1864–1869, 2001, doi: 10.1016/S0140-6736(01)06888-X.
[79] Buchert, "Ecstasy-induced reduction of the availability of the brain serotonin transporter as revealed by [11C](+)McN5652-PET and the multi-linear reference tissue model: loss of transporters or artifact of tracer kinetic modelling?," *Heterocycles*, vol. 66, no. 1, p. 7, 2007, doi: 10.3987/2005-66-0007.
[80] U. D. McCann, Z. Szabo, U. Scheffel, R. F. Dannals, and G. A. Ricaurte, "Positron emission tomographic evidence of toxic effect of MDMA ('Ecstasy') on brain serotonin neurons in human beings," *Lancet*, vol. 352, no. 9138, pp. 1433–1437, 1998, doi: 10.1016/S0140-6736(98)04329-3.
[81] M. M. L. De Win *et al.*, "Ecstasy use and self-reported depression, impulsivity, and sensation seeking: A prospective cohort study," *J. Psychopharmacol.*, vol. 20, no. 2, pp. 226–235, 2006, doi:

10.1177/0269881106063275.
[82] J. A. Lieberman and G. K. Aghajanian, “Caveat emptor: Researcher beware,” *Neuropsychopharmacology*, vol. 21, no. 4, pp. 471–473, 1999, doi: 10.1016/S0893-133X(99)00071-8.
[83] A. C. Huizink, R. F. Ferdinand, J. Van Der Ende, and F. C. Verhulst, “Symptoms of anxiety and depression in childhood and use of MDMA: Prospective, population based study,” *Br. Med. J.*, vol. 332, no. 7545, pp. 825–827, 2006, doi: 10.1136/bmj.38743.539398.3A.
[84] W. Pedersen and A. Skrondal, “Ecstasy and new patterns of drug use: A normal population study,” *Addiction*, vol. 94, no. 11, pp. 1695–1706, 1999, doi: 10.1046/j.1360-0443.1999.941116957.x.

[85] Ludewig S, Ludewig K, Hasler F, Vollenweider FX. No lasting effects of moderate doses of MDMA (Ecstasy) on memory performance and mood states in healthy humans. Biological Psychiatry 2003;53.
[86] Vollenweider FX, Gucker P, Schönbächler R, Kamber E, Vollenweider-Scherpenhuyzen MFI, Schubiger G, et al. Effects of MDMA on 5-HT uptake sites using PET and [11C]-McN5652 in humans. . Nervenarzt 2000;71
[87] Vollenweider FX, Jones RT, Baggott MJ. Caveat emptor: editors beware. Neuropsychopharmacology 2001;24:461-3.
[88] Zerssen DV. Die Beschwerden-Liste. Münchener Informationssystem. München: Psychis, 1976.
