# Supplementary material for: Oxytocin and the Role of Fluid Restriction in MDMA-Induced Hyponatremia: A Secondary Analysis of 4 Randomized Clinical Trials
Source: JAMA Netw Open. 2024 Nov 15;7(11):e2445278. doi: 10.1001/jamanetworkopen.2024.45278 (PMC11568463; doi:10.1001/jamanetworkopen.2024.45278)
Supplement: Supplement 3. — Data Sharing Statement [file jamanetwopen-e2445278-s003.pdf]

Data Sharing Statement

Atila. Oxytocin and the Role of Fluid Restriction in MDMA-Induced Hyponatremia. *JAMA Netw Open*. Published November 15, 2024. doi:10.1001/jamanetworkopen.2024.45278

Data
Additional Information: NCT04648137, NCT03019822, NCT03527316, NCT04516902.
Data available: Yes
Data types: Deidentified participant data
How to access data: We may share de-identified, individual participant-level data that underlie the results reported in this Article and related documents, including the study protocol and the statistical analysis plan. Data will be available upon publication of our main manuscript upon receipt of a request detailing the study hypothesis and statistical analysis plan. All requests should be sent to the corresponding author. Based on the scientific rigor of the proposal, the steering committee of this study will discuss all requests and decide whether data sharing is appropriate. All applicants are asked to sign a data access agreement.
When available: With publication

Supporting Documents
Document types: None

Additional Information
Who can access the data: Researchers whose proposed use of the data has been approved
Types of analyses: for research purpose
Mechanisms of data availability: after approval of a proposal
